# Frequency of Fecal Carriage of ESBL Resistance Genes in Multidrug-Resistant *Pseudomonas aeruginosa* Isolates from Cancer Patients at Laquintinie Hospital, Douala, Littoral Region, Cameroon

**DOI:** 10.1155/2024/7685878

**Published:** 2024-06-11

**Authors:** Michael F. Kengne, Armelle T. Mbaveng, Ousenu Karimo, Ballue S. T. Dadjo, Ornella D. Tsobeng, Wiliane J. T. Marbou, Victor Kuete

**Affiliations:** Department of Biochemistry, Faculty of Science, University of Dschang, Dschang, Cameroon

## Abstract

*Background*. Opportunistic infections are the second cause of death among cancer patients. This study aimed at determining the antimicrobial profile and the prevalence of extended-spectrum beta-lactamase (ESBL)-gene carriage of *Pseudomonas aeruginosa* isolates among cancer patients at the Douala Laquintinie Hospital, Littoral Region of Cameroon. Between October 2021 and March 2023, 507 study participants were recruited among whom 307 (60.55%) were cancer patients, compared to 200 (39.45%) noncancer patients. Fifty-eight *P. aeruginosa* isolates were isolated from fecal samples of forty-five cancer patients and thirteen noncancer patients using Cetrimide agar. The antimicrobial resistance profile of the isolates was determined using the Kirby–Bauer disk diffusion method. The polymerase chain reaction was used to detect the presence of extended-spectrum beta-lactamase genes among *P. aeruginosa* isolates. *P. aeruginosa* showed significant resistance rates in cancer patients compared to noncancer patients to imipenem, cefotaxime, and ceftazidime, piperacillin-tazobactam, ticarcillin-clavulanic acid, and ciprofloxacin. The multidrug resistance (MDR) rate was significantly (*p* < 0.05) higher in cancer patients than in noncancer patients. The frequency of beta-lactamase genes in the 58 ESBL-producing *P. aeruginosa* isolates was determined as 72.41% for *bla*_TEM_, 37.93% for *bla*_OXA_, 74.14% for bla_CTX‐M_, and 44.83% for *bla*_SHV_ genes. The study revealed an alarmingly high prevalence of fecal carriage of ESBL-producing *P. aeruginosa* with a high rate of MDR among cancer patients. It indicates that regular monitoring and surveillance of ESBL-producing *P. aeruginosa* among cancer patients are needed to improve the management of patients.

## 1. Background

Patients with compromised immune systems including cancer patients on chemotherapy are highly vulnerable to infections. Opportunistic infections are considered the deadliest among patients. *Pseudomonas aeruginosa* (*P. aeruginosa*) is considered one of the principal opportunistic microorganisms responsible for infections among immunocompromised patients. Opportunistic infections are the second cause of death among cancer patients [1, 2]. Infections constitute one of the main causes of mortality and morbidity in patients suffering from cancer due to the difficulty of their management caused by the emergence of multidrug-resistant (MDR) Gram-negative bacteria such as bacteria producing beta-lactamases and resistant nonfermentative rods such as *P. aeruginosa*. Treatment of bacterial infections has diminished over the past decades because of the increasing rate of antibiotic resistance leading to an increased mortality rate and this greatly interferes with anticancer therapy [3]. *P. aeruginosa* is a Gram-negative aerobic bacterium that is an important cause of nosocomial infections such as ulcerative keratitis (usually associated with contact lens wear), otitis externa (primarily in immunocompromised patients, e.g., with cancer, AIDS, and burns and those with diabetes mellitus), and skin and soft tissue infections (including diabetic foot infections) but also infections in plants, domestic, and farm animals [4, 5]. Hospitalized patients can be colonized by *P. aeruginosa* upon admission or contract *P. aeruginosa* during their hospital stay, and *P. aeruginosa* can be isolated from almost any imaginable source within hospitals [6]. The World Health Organization (WHO) considers *P. aeruginosa* among the priority pathogens, which require urgent intervention with new antimicrobial drugs because it is a serious threat due to multiresistance to many antibiotics [7]. Multidrug-resistant *P. aeruginosa* infections are responsible for high mortality rates due to their intrinsic resistance to several antimicrobial agents [8–11]. Several mechanisms are involved in the resistance of *P. aeruginosa* including overexpression of the efflux pump and acquisition of extended-spectrum *β*-lactamases (ESBLs) and metallo-*β*-lactamases (MBLs) [12]. The increased spread of bacterial resistance to antibiotics has been recognized as a global burden, particularly in developing countries [13]. Bacterial resistance to antibiotics in developing countries can be attributed to the overuse of antimicrobials in animals for food consumption, uncontrolled consumption by humans, and imperfect prescription of antibiotics by physicians [14]. This study aimed to determine the antimicrobial profile and the prevalence of ESBL gene carriage of *P. aeruginosa* isolates among cancer patients at the Douala Laquintinie Hospital, Littoral Region of Cameroon.

## 2. Materials and Methods

### 2.1. Study Area

This cross-sectional epidemiological study was conducted between October 2021 and March 2023 among consented cancer patients and noncancer patients, regardless of their age and gender, who came for consultation at the oncology unit of the Laquintinie Hospital, Douala, Littoral Region of Cameroon. Fifty-eight patients in whom the presence of *P. aeruginosa* was identified from cultured fecal samples were included in this study. Patients who were seropositive for the human immunodeficiency virus (HIV), patients on antibiotic treatment, and participants with positive serology for hepatitis B and C were excluded from this study.

### 2.2. Data Collection and Sampling Procedure

The methodology used in this study is the same as that used by Ngalani et al. (2020) [15]. Stool samples were collected from 507 patients who consented to take part in the study and from whom a bacteriological examination was requested. Duplicates were systematically eliminated. In this study, 507 stool samples were collected under aseptic conditions and processed within two hours of reception. The feces were collected by the patient as soon as they were passed, using a sterile pot supplied by us. This consisted of scraping the fecal matter with the spatula provided and placing it in a sterile pot by the patient. Patients washed their hands thoroughly with soap and water before collection. Any remaining feces were flushed down the toilet. The sample was returned to the laboratory as soon as possible for immediate microbiological analysis.

### 2.3. Isolation and Identification of *Pseudomonas aeruginosa*

Stool samples were cultured on Cetrimide agar plates, which are a differential and selective culture medium for isolation and identification of *P. aeruginosa* and incubated at 37°C for 24 h. All colonies from the primary culture were purified by subculturing onto freshly prepared Muller–Hinton agar medium and incubated at 37°C for 24–48 h. *P. aeruginosa* was identified due to its characteristic production of pyocyanin, a blue, water-soluble, nonfluorescent phenazine pigment, coupled with its colonial morphology and the characteristic grape-like odor of aminoacetophenone. We used colony-forming units (CFUs) to count the bacterial density in these samples. Further identification was performed using a series of biochemical tests including Gram-staining, an oxidase test, and observation of pyocyanin or pyoverdine production [16].

### 2.4. Antimicrobial Susceptibility Profile

Antibiotic susceptibility testing of the isolates was performed according to the Kirby–Bauer method [16]. Pure colonies of *P. aeruginosa* were inoculated into peptone water and incubated at 37°C to obtain a turbidity equal to 0.5 McFarland scale (10^8^ CFU/mL) [17–19]. A sterile swab was dipped into the inoculation, and the excess was removed by squeezing the swab against the walls of the tube. The entire surface of the Mueller–Hinton agar was swabbed. The inoculated plates were allowed to dry for 15 minutes, and antibiotic disks were deposited on the Muller–Hinton agar using sterile forceps. The plates were incubated at 37°C and examined after 18–24 h. The antimicrobial agents (Singapore BioScience PTE Ltd, Singapore) tested for *P. aeruginosa* were as follows: cefoxitin (FOX, 30 *μ*g), amoxicillin-clavulanic acid (AMC, 10 *μ*g), imipenem (IMI, 10 *μ*g), amikacin (AMI, 10 *μ*g), gentamicin (GEN, 10 *μ*g), ciprofloxacin (CIP, 5 *μ*g), ofloxacin (OFX, 5 *μ*g), trimethoprim-sulfamethoxazole (COT, 10 *μ*g), tetracycline (TET, 10 *μ*g), nitrofurantoin (NIT, 30 *μ*g), nalidixic acid (NAL, 30 *μ*g), cefotaxime (CTX, 5 *μ*g), cefuroxime (CXM, 30 *μ*g), ceftazidime (CAZ, 10 *μ*g), piperacillin (PRL, 30 *μ*g), piperacillin-tazobactam (PPT, 30 *μ*g), and ticarcillin-clavulanic acid (TTC, 75 *μ*g). *E. coli* American-Type Culture Collection (ATCC) 25922 maintained in the Research Unit of Microbiology and Antimicrobial Substances Laboratory was used as positive control. Results were expressed as follows: sensitive (S), intermediate (I), and resistant (R) based on the recommendations of the Clinical and Laboratory Standards Institute [20]. Bacteria were considered multidrug-resistant if they were resistant to at least three antibiotics of three different classes or families [21, 22].

### 2.5. DNA Extraction

DNA extraction from fresh colonies was carried out by the heat shock method. A loop full of fresh bacterial colonies was suspended in 400 *μ*L of 1X Tris-EDTA buffer (0.1 M Tris-Cl and 0.01 M EDTA diluted 1/10). The suspension was vortexed for 5 seconds and heated in a water bath at 95°C for 25 minutes. The heated suspension was centrifuged at 13,000 rpm for 3 minutes. The supernatant fluid containing the DNA was diluted tenfold and stored at −20°C for molecular analyses by polymerase chain reaction (PCR) [23].

### 2.6. Screening for ESBL Genes

The PCR was run for the detection of the following ESBL genes: *bla*_TEM_, *bla*_OXA_, bla_CTX‐M_, and *bla*_SHV_. The required concentrations of the different components and the specific final volumes of the mixture were pipetted into the individual PCR tubes as required. In brief, 14.9 *μ*L of deionised Sigma water (New England Biolabs, Ipswich, MA) was dispensed into each PCR tube and the following was added: master mix (2.5 *μ*L buffer + 2 mM MgCl_2_ + 0.5 *μ*L dNTP mix + 20 *μ*M forward primer (1.0 *μ*L) + 20 *μ*M reverse primer (1.0 *μ*L) + 0.1 *μ*L standard taq DNA polymerase 5. 0 U (New England Biolab, UK) were added to a 1.5 ml tube). These volumes were multiplied by the number of samples to be analysed. 20 *μ*L of the master mix was pipetted into each PCR tube and 5.0 *μ*L of DNA sample was then added to each PCR tube. The PCR tubes were then inserted into a Techne® thermal cycler [23].  Table 1 shows the primer sequences used and PCR conditions as described by a previous study with slight modifications [24]. The amplified products were then subjected to electrophoresis in a 1.5% agarose gel and visualized using a transilluminator.

### 2.7. Ethical Considerations

Ethical approvals were obtained from the National Committee for Ethics in Human Sciences Research (CNERSH), Yaoundé-Cameroon (no. N°2022/09/129/CE/CNERSH/SP) and from the Institutional Ethics Committee of the University of Douala (CIE-UD) (No. 3127/CEI-UDo/06/2022/T).

### 2.8. Processing and Statistical Analysis of Data

The susceptibility profile was expressed as a percentage. The chi-square test was used to compare the resistance frequencies of *P. aeruginosa* in cancer and noncancer patients. A *p* < 0.05 was considered significant. The visual dashboard test was used to compare the 95% odds ratios of the ESBL gene in *P. aeruginosa,* to infer a possible relationship between the resistance profile and the resistance genes. All these analyses were carried out using Epi InfoTM software version 7.2.4 (CDC, 1600 Clifton Road Atlanta, GA 303294027 USA).

## 3. Results

### 3.1. Distribution of *Pseudomonas aeruginosa* Isolated according to Different Age Groups

**Figure 1:**
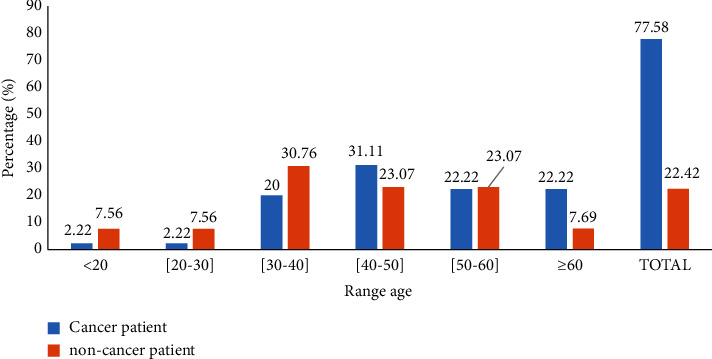
Distribution of *Pseudomonas aeruginosa* isolated according to different age groups.

Of 507 participants, *P. aeruginosa* was isolated in 58 (11.43%) participants, that is, 45 (77.58%) from cancer patients and 13 (22.42%) among noncancer patients (Figure 1). On the other hand, more *P. aeruginosa* was isolated in cancer and noncancer patients in the age groups of 25–34 years (31.11% and 23.07%) and 51–60 years (22.22% and 23.07%), respectively.

### 3.2. Antibiotic Resistance Profile of *Pseudomonas aeruginosa* Isolates from Cancer and Noncancer Patients

Susceptibility of *P. aeruginosa isolates* was tested using nineteen different antibiotics during this study.  Table 2 presents the susceptibility results of *P. aeruginosa* isolates to these antibiotics. *P. aeruginosa* isolates presented significantly (*p* < 0.05) high resistance rates in cancer patients compared to noncancer patients. These resistance rates were as follows: AMX (97.78% versus 84.62%), IMP (82.22% versus 30.77%), CTX (97.78% versus 84.68%), FOX (95.56% versus 69.23%), CAZ (100% versus 69.23%), PPT (100% versus 69.23%), CBT (100% versus 84.62%), CIP (93.33% versus 7.69%), and NAL (97.78% versus 7.69%). On the other hand, the resistance rates of *P. aeruginosa* towards AMK (6.67% versus 30.77%) and GEN (8.89% versus 61.54%) were lower in participants with cancer compared to noncancer participants.

### 3.3. Frequency of Multidrug-Resistant (MDR) *Pseudomonas aeruginosa* Isolates

**Figure 2:**
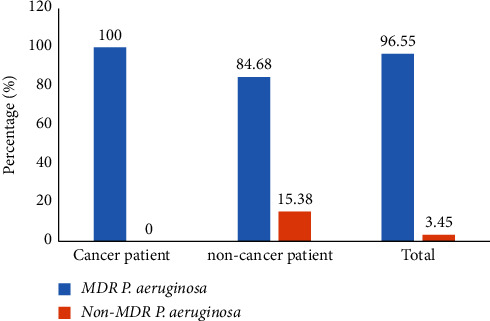
Frequency of appearance of multidrug-resistant (MDR) *Pseudomonas aeruginosa* among cancer and noncancer patients.

Figure 2 shows the frequency of MDR in different isolates among cancer and noncancer participants. The prevalence of MDR of *P. aeruginosa* was 96.55%. MDR *P. aeruginosa* isolates were significantly ((100% versus 84.62%; *p* < 0.05) elevated in patients suffering from cancer compared to noncancer patients.

### 3.4. Effect of Anticancer Treatment on the Resistance Profile of *Pseudomonas aeruginosa*

**Figure 3:**
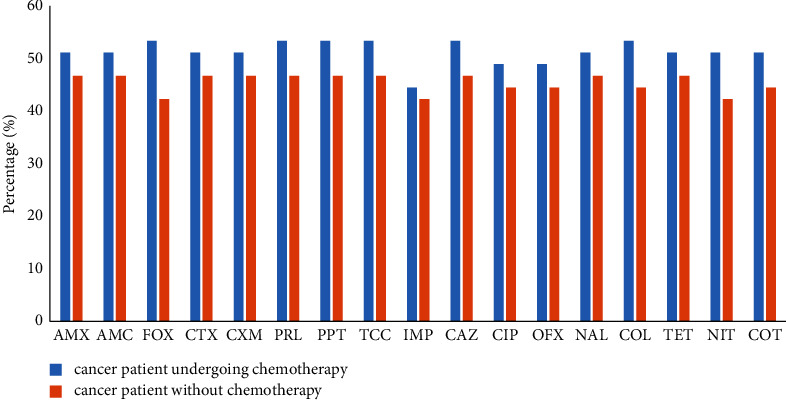
Effect of anticancer treatment on the resistance profile of *Pseudomonas aeruginosa*. IPM: imipenem, AMX: amoxicillin, AMC: amoxicillin-clavulanic acid, CAZ: ceftazidime, FOX: cefoxitin, CTX: cefotaxime, CXM: cefuroxime, PRL: piperacillin, TET: tetracycline, CIP: ciprofloxacin, OFX: ofloxacin, NAL: nalidixic acid, COT: trimethoprim-sulfamethoxazole, COL: colistin, NIT: nitrofurantoin, PPT: piperacillin-tazobactam, and TCC: ticarcillin-clavulanic acid.

Figure 3 shows the resistance profile of *P. aeruginosa* isolates based on anticancer therapy. The figure shows high resistance rates in cancer patients who underwent courses of chemotherapy compared to cancer patients without cancer treatment.

### 3.5. Genotypic Detection of ESBL Carriage

**Figure 4:**
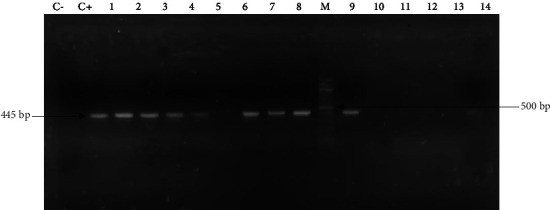
Gel picture showing the amplification of the *bla*_TEM_ gene fragment (445 bp). C−: negative control, C+: positive control, and M: molecular weight marker (100 bp ladder). The lines 1 to 4 and 6 to 8 and 9 are positive isolates containing the *bla*_TEM_.

**Figure 5:**
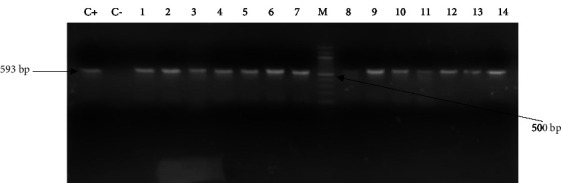
Gel picture showing the amplification of the bla_CTX‐M_ type gene fragment (593 bp). C−: negative control, C+: positive control, and M: molecular weight marker (100 bp ladder). The lines 1 to 14 are positive isolates containing the bla_CTX‐M_.

**Figure 6:**
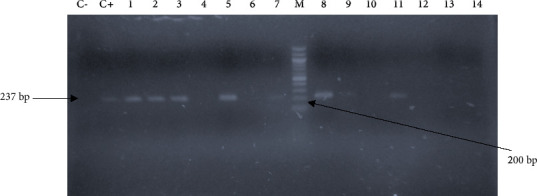
Gel picture showing the amplification of the *bla*_SHV_ gene fragment (237 bp). C−: negative control, C+: positive control, and M: molecular weight marker (100 bp ladder). The lines 1 to 3, 5 and 7 to 9, and 11 are positive isolates containing the *bla*_SHV_.

**Figure 7:**
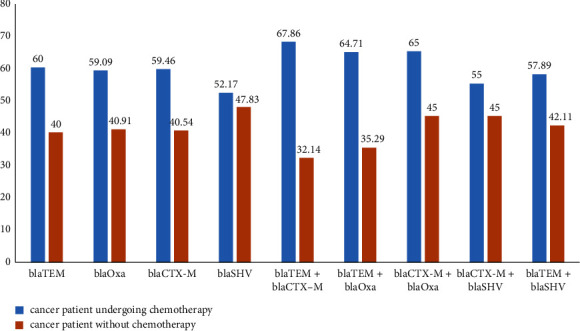
Effect of anticancer treatment on the ESBL genes of *Pseudomonas aeruginosa*.

Of the 58 isolates screened for ESBL production, 94.83% (55/58) harbored at least one ESBL gene. The most prevalent gene detected in this study was the bla_CTX‐M_ type gene (74.14%, 43/58) followed by *bla*_TEM_ (72.41%, 42/58) and the least gene detected was *bla*_OXA_(37.93%, 22/58). More than half (74.14%, 43/58) of the isolates harbored more than one ESBL gene (Table 3). Figures 4–6 present gel pictures of bla_TEM_, bla_CTX‐M_, and bla_SHV_, respectively.  Table 4 shows *P. aeruginosa* resistance genes in the different groups. The results showed that the *bla*_*TEM*_ gene was higher (*p*=0.090) in *P. aeruginosa* isolated from cancer patients (86.05%) compared to those isolated from noncancer patients (13.95%), while *bla*_OXA_ was significantly higher (*p*=0.001) in *P. aeruginosa* isolated from cancer patients (100.00%) compared to those isolated from noncancer patients (0. 00%). The results also show that the bla_CTX‐M_ type gene was significantly higher (*p*=0.0001) in *P. aeruginosa* isolated from cancer patients (86.05%) compared to those isolated from noncancer patients (13. 95%) and the *bla*_SHV_ gene was significantly higher (*p*=0.008) in *P. aeruginosa* isolated from cancer patients (88.46%) compared to those isolated from noncancer patients (11.54%).  Figure 7 shows the molecular profile of resistance genes among *P. aeruginosa* isolates depending on cancer treatment.  Figure 7 shows high ESBL gene rates in cancer patients who underwent courses of chemotherapy compared to cancer patients without cancer treatment. Evaluation of the correlation of carriage of the resistance genes: bla_TEM_, bla_OXA_, bla_CTX‐M_ type gene, and *Bla*_SHV_ with antibiotic resistance in *P. aeruginosa* isolates is shown in Table 5. There was a significant correlation between carriage of the *bla*_TEM_ gene in *P. aeruginosa* and resistance to antibiotics against CAZ (*p*=0.032); carriage of the bla_CTX‐M_ type gene and resistance to PPT (*p*=0.0262), CAZ (*p*=0.0262) in *P. aeruginosa*. Carriage of *bla*_OXA_ resistance genes was found to pose a higher risk for *P. aeruginosa* resistance to IMP (odds ratio (OR) = 2.541); *bla*_SHV_ resistance genes with a higher risk for resistance to FOX (odds ratio (OR) = 4.622), PPT (odds ratio (OR) = 2.568); bla_CTX‐M_ resistance genes with higher risk for resistance to CTX (odds ratio (OR) = 6.461), FOX (odds ratio (OR = 3.333), and AMX (odds ratio (OR) = 6.461).

Evaluation of the correlation of carriage of the resistance genes bla_TEM_+bla_CTX‐M_, bla_TEM_+bla_OXA_, bla_TEM_+bla_SHV_, bla_CTX‐M_+bla_OXA_, and bla_CTX‐M_+bla_SHV_ with antibiotic resistance in *P. aeruginosa* isolates is shown in Table 6. Simultaneous carriage of two resistance genes bla_TEM_+bla_CTX‐M_ was found to pose a higher risk for *P. aeruginosa* resistance to IMP (OR = 2.511), AMX (OR = 2.401), FOX (OR = 2.521), and PPT (OR = 3.751); of two resistance genes b*la*_TEM_+*bla*_SHV_ with higher risk for resistance to FOX (OR = 3.125) and bla_CTX‐M_+bla_SHV_ with higher risk for resistance to FOX (OR = 3.793) and PPT (OR = 2.120).

## 4. Discussion

Treatment of bacterial infections has been impaired due to the alarming rate of resistance that keeps on increasing with time especially those responsible for nosocomial and opportunistic infections [2]. The bacteria frequently responsible for nosocomial and opportunistic infections in Cameroon and the city of Douala are Gram-negative bacilli, including *P. aeruginosa*, which cause infection in immunocompromised patients [25]. The prevalence of *P. aeruginosa* is estimated to be between 7.1% and 7.3% among all nosocomial infections [26]. *P. aeruginosa* is a particularly important pathogen in immunocompromised patients, particularly in patients with neutropenia. It is a critically important pathogen in patients with hematological malignancies, while also showing an increasing trend of MDR isolates in these patients [27, 28]. *P. aeruginosa* has now emerged as an ever-increasing problem due to its increasing resistance to several antibiotics. This study determined the susceptibility profile of *P. aeruginosa* isolates isolated from the fecal samples of cancer and noncancer patients at the Laquintinie Hospital in Douala to provide physicians with up-to-date information on local resistance data and antimicrobials for this pathogen.

Of the 507 participants, *P. aeruginosa* was isolated from 11.43% which is 77.58% from cancer patients and 22.42% from noncancer patients. On the other hand, more *P. aeruginosa* was isolated *from* cancer and noncancer patients belonging to the age groups of 40–49 years (31.11% and 23.07%) and 50–59 years (22 .22% and 23.07%), respectively. This result is higher than the 6.7% reported in an Indian study [29]. This difference in prevalence could be due to differences in sample size and immunity of participants. *P. aeruginosa isolates exhibited significantly (p* < 0.05) high resistance rates in cancer patients compared to noncancer patients: AMX (97.78% vs. 84.62%), IMP (82.22% versus 30.77%), CTX (97.78% versus 84.68).%), FOX (95.56% against 69.23%), CAZ (100% against 69.23%), PPT (100% against 69.23%), CBT (100% against 84.62%), CIP (93.33% versus 7.69%), and NAL (97.78% versus 7.69%). Contrary to the above, resistance rates of *P. aeruginosa* AMK (6.67% vs. 30.77%) and GEN (8.89% vs. 61.54%) were lower in participants with cancer than in those without cancer. These results show a marked increase in resistance of *P. aeruginosa* isolates in cancer patients compared to noncancer patients. Considering these results, the resistance of *P. aeruginosa* to beta-lactam antibiotics was very high. Garg et al. also found high levels of resistance to beta-lactam antibiotics in their study [30]. The increasing rate of Gram-negative bacilli towards cephalosporins could be explained by the fact that these antibiotics are commonly used to treat infections caused by the later inducing drug pressure. *P. aeruginosa* isolates were also highly resistant to quinolones and fluoroquinolones. The very high rates of quinolone resistance observed in our work could be explained by the fact that exposure to fluoroquinolones is a risk factor for MDR Gram-negative bacilli infection in cancer patients [31]. Frequent consumption of antibiotics without medical prescription in our community can lead to selective pressure of antibiotics on *P. aeruginosa* making this organism change its resistant mechanisms. We found an overall high rate of multidrug resistance among *P. aeruginosa* isolates and, importantly, a significant increase was observed in cancer patients compared to noncancer patients. These results are consistent with other reports on patients with hematological malignancies [32, 33]. The emergence of resistance among *P. aeruginosa* isolates causing infection in neutropenic patients can be attributed to the administration of inadequate empiric antibiotic therapy, which severely impairs patient outcomes [33]. This high rate of multiresistance as well as the high rate of resistance observed in cancer patients undergoing anticancer chemotherapy can be explained by the action of anticancer chemotherapy. Certain cytotoxic agents used in cancer treatment have antibacterial properties and have an impact on increasing bacterial mutation rates by activating the SOS response [34–36]. This therefore contributes to the spread of antibiotic resistance mechanisms in bacterial populations in response to stress [37]. Cancer patients are treated with substances that can modify bacterial DNA and increase bacterial mutation rates, leading to antibiotic resistance by accelerating the expansion of KPC-type *β*-lactamases (*Klebsiella pneumoniae* carbapenemase) [38–41].

*P. aeruginosa* is a pathogenic bacterium, causing nosocomial infections with intrinsic and acquired resistance mechanisms to a large group of antibiotics, including *β*-lactams. Cameroon is currently experiencing an increase in the number of bacterial infections associated with a broad spectrum of resistance to common antimicrobial agents. The presence of ESBL-producing bacteria was reported in this study. Molecular analysis revealed that bla_CTX‐M_ is the most prevalent (74.14%) ESBL gene followed by *bla*_TEM_, which was present in 72.41% of resistant strains. The reason behind this increased frequency of occurrence of ESBL-producing organisms is likely due to the misuse and abuse use of antibiotics [42]. It seems that the bla_CTX‐M_ type gene is the predominant genotype worldwide, particularly in Enterobacteriaceae [43]. Few studies from other regions of the world have shown different prevalences of the bla_CTX‐M_ type gene among isolates, including 84.7% (Chile) and 98.8% (China) [44, 45]. We observed that the *bla*_SHV_ and *bla*_OXA_ genes were less common in our contexts, with a respective prevalence of 44.83% and 37.33%. A report from Hamad Medical Corporation, Qatar stated that the bla_CTX‐M_ type gene evolved through mutations in the *bla*_TEM_ and *bla*_SHV_ genes and is recently endemic [46].

This phenomenon may pose serious risks to public health, as it would result in substantial limitations of therapeutic options. Thus, appropriate control measures, including establishing screening strategies to identify ESBL-producing bacteria, are necessary to prevent these strains from spreading. CTX-M was the most prevalent gene detected in ESBL-producing *P. aeruginosa* in the present study. This is consistent with a study conducted in the eastern region of Saudi Arabia, in which bla_CTX‐M_ type gene (97.4%) was more common than *bla*_SHV_ (23.1%) and *bla*_TEM_ (0.0%) [47]. Likewise, studies in the eastern region have reported the predominance of the bla_CTX‐M_ type gene in Gram-negative bacteria producing ESBLs [48, 49]. As an illustration, bla_CTX‐M_ type gene was the most common type in the Asia-Pacific region, followed by *bla*_SHV_ and *bla*_TEM_ [49]. In Nigeria, the most frequent gene types among isolates from patients with surgical site infections were bla_SHV_, bla_CTX‐M_, and bla_OXA_ [50]. In Burkina Faso, the most prevalent ESBL resistance genes were bla_CTX‐M_ (40.1%), *bla*_TEM_ (26.2%), and *bla*_SHV_ (5.9%) in *Enterobacteriaceae* [51]. These results, together with the present findings, revealed that the prevalence of ESBL gene types might vary from one geographical location to the other.

Although this study presents data on cancer patients with limited information on the resistance of *P. aeruginosa* to commonly used antibiotics, some limitations must be acknowledged. The generalizability of the data could be compromised by sampling bias. Furthermore, the data should not be generalised to the whole country. The study showed the prevalence of fecal carriage of ESBL-producing P. aeruginosa with a high rate of MDR in cancer patients however sequencing of the ESBLs studied in this work would be important in order to confirm TEM, SHV, or OXA ESBLs from other variants which are simple penicillinases. Despite the limitations mentioned, we remain convinced that this study provides essential information on the intestinal carriage of multidrug-resistant *P. aeruginosa* in cancer patients. Furthermore, plasmids are involved in gene transfer and carry additional antibiotic resistance genes as well as *β*-lactam resistance genes. These data therefore have important implications for the quality of patient care and infection control practices.

## 5. Conclusion

The study revealed an alarmingly high prevalence of fecal carriage of ESBL-producing *P. aeruginosa* with a high rate of MDR among cancer patients. bla_CTX‐M_ type gene was the most prevalent gene detected. Furthermore, the coexistence of two different ESBL genes was frequently detected in this bacterial pathogen. Regular monitoring and surveillance in the screening of ESBL-producing *P. aeruginosa* among cancer patients are needed to improve the management of patients.

## Figures and Tables

**Table 1:** Specific PCR primers used in this study for the determination of antibiotic resistance genes.

ESBL gene	Primers	Sequence (5′-3′)	Amplicon size (bp)	PCR conditions (35 cycles)	Reference
*bla*_TEM_	*bla*_TEM_-F	5′CGCCGCATACACTATTCTCAGAATGA3′	445	7 minutes at 94°C, 30 seconds at 94°C, 30 seconds at 58°C, 10 minutes at 68°C, and 1 minute at 68°C	[19]
	*bla*_TEM_-R	5′ACGCTCACCGGCTCCAGATTTAT-3′			
*bla*_OXA_	*bla*_OXA_-F	5′ACACAATACATATCAACTTCGC3′	813	7 minutes at 94°C, 30 seconds at 94°C, 30 seconds at 58°C, 10 minutes at 68°C, and 1 minute at 68°C	
	*bla*_OXA_-R	5′-AGTGTGTTTAGA ATGGTGATC-3′			
*bla*_CTXM_	*bla*_CTXM_-F	5′-ATGTGCAGYACCAGTAARGTKATGGC3′	593	7 minutes at 94°C, 30 seconds at 94°C, 30 seconds at 55°C, 10 minutes at 68°C, and 1 minute at 68°C	
	*bla*_CTXM_-R	5′-TGGGTRAARTARGTSACCAGAAYCAGCGG-3′			
*bla*_SHV_	*bla*_SHV_-F	5′-CTTTATCGGCCCTCA CTCAA-3′	237	7 minutes at 94°C, 30 seconds at 94°C, 30 seconds at 62°C, 10 minutes at 68°C, and 1 minute at 68°C	
	*bla*_SHV_-R	5′-AGGTGCTCATCATGGGAAAG-3′			

**Table 2:** Antibiotic resistance profile of *Pseudomonas aeruginosa* isolates from cancer and noncancer patients.

Antibiotics		*Pseudomonas aeruginosa*			
		Cancer+ *n* = 45 (%)	Cancer− *n* = 13 (%)	*x* ^2^	*P* value
IMP	S	2 (4.44)	0 (0.00)	16.54	**<0.001**
	I	6 (13.33)	9 (69.23)		
	R	37 (82.22)	4 (30.77)		

AMX	S	1 (2.22)	0 (0.00)	7.39	**0.021**
	I	0 (0.00)	2 (15.38)		
	R	44 (97.78)	11 (84.62)		

AMC	S	1 (2.22)	0 (0.00)	1.21	**0.452**
	I	0 (0.00)	0 (0.00)		
	R	44 (97.78)	13 (100)		

FOX	S	2 (4.44)	0 (0.00)	15.20	**<0.001**
	I	0 (0.00)	4 (30.77)		
	R	43 (95.56)	9 (69.23)		

CTX	S	1 (2.22)	0 (0.00)	7.39	**0.024**
	I	0 (0.00)	2 (15.38)		
	R	44 (97.78)	11 (84.68)		

CXM	S	1 (2.22)	0 (0.00)	3.78	0.150
	I	0 (0.00)	1 (7.69)		
	R	44 (97.78)	12 (92.31)		

PRL	S	0 (0.00)	0 (0.00)	0.44	0.112
	I	0 (0.00)	1 (7.69)		
	R	45 (100)	12 (92.31)		

PPT	S	0 (0.00)	1 (7.69)	14.87	<0.001
	I	0 (0.00)	3 (23.08)		
	R	45 (100)	9 (69.23)		

CAZ	S	0 (0.00)	4 (30.77)	10.46	<0.001
	R	45 (100)	9 (69.23)		

TCC	S	0 (0.00)	0 (0.00)	7.17	0.020
	I	0 (0.00)	2 (15.38)		
	R	45 (100)	11 (84.62)		

AMK	S	42 (93.33)	9 (69.23)	20.39	<0.001
	R	3 (6.67)	4 (30.77)		

GEN	S	41 (91.11)	5 (38.46)	13.98	<0.001
	R	4 (8.89)	8 (61.54)		

CIP	S	2 (4.44)	1 (7.69)	45.27	<0.001
	I	1 (2.22)	11 (84.62)		
	R	42 (93.33)	1 (7.69)		

OFX	S	2 (4.44)	1 (7.69)	43.27	<0.001
	I	1 (2.22)	11 (84.62)		
	R	42 (93.33)	1 (7.69)		

NAL	S	1 (2.22)	1 (7.69)	49.50	<0.001
	I	0 (0.00)	11 (84.62)		
	R	44 (97.78)	1 (7.69)		

COL	S	1 (2.22)	0 (0.00)	3.78	0.150
	I	0 (0.00)	1 (7.69)		
	R	44 (97.78)	12 (92.31)		

TET	S	1 (2.22)	0 (0.00)	3.78	0.150
	I	0 (0.00)	1 (7.69)		
	R	44 (97.78)	12 (92.31)		

NIT	S	3 (6.67)	0 (0.00)	4.33	0.115
	I	0 (0.00)	1 (7.69)		
	R	42 (93.33)	12 (92.31)		

COT	S	2 (4.44)	0 (0.00)	1.25	0.594
	R	43 (95.56)	10 (100)		

IPM, imipenem; AMX, amoxicillin; AMC, amoxicillin -clavulanic acid CAZ, ceftazidime; FOX, cefoxitin; CTX, cefotaxime; CXM, cefuroxime; PRL, piperacillin; AMK, amikacin; GEN, gentamicin; TET, tetracycline; CIP, ciprofloxacin; OFX, ofloxacin; NAL, nalidixic acid; COT, trimethoprim-sulfamethoxazole; COL, colistin; NIT, nitrofurantoin; PPT, piperacillin-tazobactam; TCC, ticarcillin-clavulanic acid. In bold: positive association between the antibiotic and the virulent gene.

**Table 3:** Extended-spectrum *β*-lactamase (ESBL) gene types detected in clinical isolates of *P. aeruginosa.*

Positive by PCR for ESBL genes	Number amplified (*N* = 58)	Percentage
Single ESBL gene		
*bla*_TEM_	42	72.41
*bla*_OXA_	22	37.93
bla_CTX‐M_	43	74.14
*bla*_SHV_	26	43.48
Two or more ESBL genes		
*bla*_TEM_ *+* bla_CTX‐M_	31	53.45
*bla*_TEM_ *+* *bla*_OXA_	17	29.31
*bla*_TEM_ *+* *bla*_SHV_	21	36.21
bla_CTX‐M_ *+* *bla*_OXA_	20	34.48
bla_CTX‐M_ *+* *bla*_SHV_	23	39.66

**Table 4:** Frequency of ESBL genes among *Pseudomonas aeruginosa* isolates based on cancer status.

ESBL genes	Cancer patients *N* = 45 (%)	Noncancer patients *N* = 13 (%)	*x* ^2^	*p* value
Single ESBL gene				
*bla*_TEM_	35 (83.33)	7 (16.67)	2.89	0.090
*bla*_OXA_	22 (100)	0 (0.00)	10.23	0.001
bla_CTX‐M_	37 (86.05)	6 (13.95)	19.36	0.001
*bla*_SHV_	23 (88.46)	3 (11.54)	19.06	0.001
Two or more ESBL genes				
*bla*_TEM_ *+* bla_CTX‐M_	28 (90.32)	3 (9.65)	6.21	0.014
*bla*_TEM_ *+* *bla*_OXA_	17 (100)	0 (0.00)	6.94	0.005
*bla*_TEM_ *+* *bla*_SHV_	19 (90.48)	2 (9.52)	3.14	0.070
bla_CTX‐M_ *+* *bla*_OXA_	20 (100)	0 (0.00)	8.81	0.001
bla_CTX‐M_ *+* *bla*_SHV_	20 (86.96)	3 (23.04)	1.92	0.091

**Table 5:** Correlation of resistance genes and *Pseudomonas aeruginosa* resistance to antibiotics of the *β*-lactam family.

Antibiotic-resistant Patterns	*bla*_TEM_		*bla*_OXA_		bla_CTX‐M_		*bla*_SHV_	
	Odds ratio (LI-UI)	*P* value	Odds ratio (LI-UI)	*P* value	Odds ratio (LI-UI)	*P* value	Odds ratio (LI-UI)	*P* value
AMX	1.333 (0.112–15.806)	0.404	1.230 (0.105–14.478)	0.455	6.461 (0.541–77.140)	0.087	1.666 (0.142–19.472)	0.369
AMC	NA	NA	NA	0.189	NA	0.370	NA	0.275
FOX	1.357 (0.223–8.248)	0.110	1.250 (0.209–7.462)	0.421	3.333 (0.593–18.717)	0.103	4.622 (0.505–42.410)	0.087
CTX	1.333 (0.112–15.806)	0.404	NA	0.115	6.461 (0.541–77.143)	0.047	1.666 (0.142–19.478)	0.369
CXM	NA	0.115	NA	0.190	NA	0.273	NA	0.190
PRL	NA	NA	NA	0.310	NA	0.129	NA	0.275
PPT	0.866 (0.083–8.999)	0.480	NA	0.069	10.500 (0.999–110.361)	**0.0262**	2.586 (0.252–26.463)	0.236
CAZ	9.460 (0.904–98.974)	**0.032**	NA	0.069	10.501 (0999–110.360)	**0.0262**	NA	0.042
TCC	NA	NA	NA	0.190	NA	0.031	NA	0.150
IMP	2.488 (0.737–8.401)	0.079	2.541 (0.707–9.140)	0.079	1.291 (0.365–4.569)	0.347	1.233 (0.392–3.874)	0.366

LI, lower interval; UI, upper interval. The *p* value is given at 95% CI and significant at ≤0.05. NA, not applicable. IPM, imipenem; AMX, amoxicillin; AMC, amoxicillin-clavulanic acid CAZ, ceftazidime; FOX, cefoxitin; CTX, cefotaxime; CXM, cefuroxime; PRL, piperacillin; PPT, piperacillin-tazobactam; TCC, ticarcillin-clavulanic acid; in bold, positive association between antibiotic and the resistance gene.

**Table 6:** Correlation of two ESBL genes and *Pseudomonas aeruginosa* resistance to *β*-lactam antibiotics.

Antibiotic-resistant Patterns	*bla*_TEM_ *+* bla_CTX‐M_		*bla*_TEM_ *+* *bla*_OXA_		*bla*_TEM_ *+* *bla*_SHV_		bla_CTX‐M_ *+* *bla*_OXA_		bla_CTX‐M_ *+* *bla*_SHV_	
	Odds ratio (LI-UI)	*P* value	Odds ratio (LI-UI)	*P* value	Odds ratio (LI-UI)	*p* value	Odds ratio (LI-UI)	*P* value	Odds ratio (LI-UI)	*p* value
AMX	**2.401** (0.205–28.048)	0.271	0.820 (0.0694–9.700)	0.428	1.142 (0.097–13.411)	0.478	1.055 (0.089–12.404)	0.498	1.374 (0.113–13.112)	0.425
AMC	NA	0.534	NA	0.146	NA	0.318	NA	0.172	NA	0.298
FOX	**2.521** (0.423–15.005)	0.169	0.810 (0.134–4.906)	0.404	**3.125** (0339–28.731)	0.170	1.058 (0.176–6.348)	0.489	**3.793** (0.413–34.834)	0.125
CTX	NA	0.047	NA	0.172	1.142 (0.097–13.411)	0.478	NA	0.136	NA	0.102
CXM	NA	0.140	NA	0.248	0.556 (0.032–9.369)	0.362	NA	0.212	0.666 (0.039–11.227)	0.403
PRL	NA	0.232	NA	0.353	NA	0.318	NA	0.212	NA	0.298
PPT	**3.751** (0.366–38.388)	0.148	NA	0.119	1.761 (0.171–18.130)	0.347	NA	0.086	**2.120** (0.207–21.843)	0.291
CAZ	**10.142** (0.960–110.123)	**0.020**	NA	0.118	NA	0.077	NA	0.085	NA	0.058
TCC	NA	0.106	NA	0.248	NA	0.201	NA	0.212	NA	0.175
IMP	**2.511** (0.639–6.359)	0.123	**2.411** (0.594–9.853)	0.113	1.057 (0.324–3.448)		2.080 (0.575–7.514)	0.139	1.355 (0.418–4.390)	0.315

LI, lower interval; UI, uppper interval. The *p*p value is given at 95% CI and significant at ≤0.05. NA, not applicable. IPM, imipenem; AMX, amoxicillin; AMC, amoxicillin-clavulanic acid CAZ, ceftazidime; FOX, cefoxitin; CTX, cefotaxime; CXM, cefuroxime; PRL, piperacillin; PPT, piperacillin-tazobactam; TCC, ticarcillin-clavulanic acid; in bold, positive association between antibiotic and the resistance gene.

## Data Availability

The data used to support the findings of this study are included within the article.

## Abbreviations

AMC: Amoxicillin-clavulanic acid
AMI: Amikacin
ATCC: American-Type Culture Collection
CAZ: Ceftazidime
CFU: Colony-forming unit
CIP: Ciprofloxacin
CNERSH: National Committee for Ethics in Human Sciences Research
COT: Trimethoprim-sulfamethoxazole
CTX: Cefotaxime
CXM: Cefuroxime
EDTA: Ethylenediaminetetraacetic acid
ESBL: Extended-spectrum beta-lactamase
FOX: Cefoxitin
GEN: Gentamicin
HIV: Human immunodeficiency virus
IMI: Imipenem
KPC: *Klebsiella pneumoniae* carbapenemase
MBL: Metallo-*β*-lactamases
MDR: Multidrug-resistant
NAL: Nalidixic acid
NIT: Nitrofurantoin
OFX: Ofloxacin
PCR: Polymerase chain reaction
PPT: Piperacillin-tazobactam
PRL: Piperacillin
TET: Tetracycline
TTC: Ticarcillin-clavulanic acid
WHO: World Health Organization.

## Supplementary Materials

Supplementary Materials All raw data generated to support the results of this study are available in the following: S1: general information on the study population; S2: detection of *Pseudomonas aeruginosa* and antibiogram; and S3: detection of resistance genes.

## References

[1] Nanayakkara A. K., Boucher H. W., Fowler V. G., Jezek A., Outterson K., Greenberg D. E. (2021). Antibiotic resistance in the patient with cancer: Escalating challenges and paths forward. *CA: A Cancer Journal for Clinicians*.

[2] Fongang H., Mbaveng A. T., Kuete V. (2023). Chapter one-global burden of bacterial infections and drug resistance. *Advances in Botanical Research*.

[3] Perez F., Adachi J., Bonomo R. A. (2014). Antibiotic-resistant Gram-negative bacterial infections in patients with cancer. *Clinical Infectious Diseases*.

[4] Bonten M. J., Bergmans D. C., Speijer H., Stobberingh E. E. (1999). Characteristics of polyclonal endemicity of *Pseudomonas aeruginosa* colonization in intensive care units. Implications for infection control. *American Journal of Respiratory and Critical Care Medicine*.

[5] Crone S., Vives-Flórez M., Kvich L. (2020). The environmental occurrence of *Pseudomonas aeruginosa*. *Apmis*.

[6] Pirnay J. P., De Vos D., Cochez C. (2003). Molecular epidemiology of *Pseudomonas aeruginosa* colonization in a burn unit: persistence of a multidrug-resistant clone and a silver sulfadiazine-resistant clone. *Journal of Clinical Microbiology*.

[7] Tacconelli E. (2017). Global priority list of antibiotic-resistant bacteria to guide research, discovery, and development. *World Health Organ*.

[8] Voukeng I. K., Beng V. P., Kuete V. (2016). Antibacterial activity of six medicinal Cameroonian plants against Gram-positive and Gram-negative multidrug resistant phenotypes. *BMC Complementary and Alternative Medicine*.

[9] Omosa L. K., Midiwo J. O., Mbaveng A. T. (2016). Antibacterial activities and structure–activity relationships of a panel of 48 compounds from k. *Springer Plus*.

[10] Dzotam J. K., Simo I. K., Bitchagno G. (2018). *In vitro* antibacterial and antibiotic modifying activity of crude extract, fractions and 3′,4′,7-trihydroxyflavone from *Myristica fragrans* Houtt against MDR Gram-negative enteric bacteria. *BMC Complementary and Alternative Medicine*.

[11] Farhan S. M., Ibrahim R. A., Mahran K. M., Hetta H. F., Abd El-Baky R. M. (2019). Antimicrobial resistance pattern and molecular genetic distribution of metallo-*β*-lactamases producing *Pseudomonas aeruginosa* isolated from hospitals in Minia, Egypt. *Infection and Drug Resistance*.

[12] Chaudhary M., Payasi A. (2013). Rising antimicrobial resistance of Pseudomonas aeruginosa isolated from clinical specimens in India. *Journal of Proteomics & Bioinformatics*.

[13] Chellapandi K., Dutta T. K., Sharma I., De Mandal S., Kumar N. S., Ralte L. (2017). Prevalence of multi drug resistant enteropathogenic and enteroinvasive Escherichia coli isolated from children with and without diarrhea in Northeast Indian population. *Annals of Clinical Microbiology and Antimicrobials*.

[14] Mouiche M. M. M., Moffo F., Akoachere J. F. T. K. (2019). Antimicrobial resistance from a one health perspective in Cameroon: A systematic review and meta-analysis. *BMC Public Health*.

[15] Ngalani O. J. T., Marbou W. J. T., Mbaveng A. T., Kuete V. (2020). Resistance profiles of Staphylococcus aureus and immunological status in pregnant women at bafang, west region of Cameroon: a cross-sectional study: A cross-sectional study. *Cureus*.

[16] Park Talaro K., Cowan M. K., Chess B. (2009). *Foundations in Microbiology*.

[17] Kuete V., Wansi J. D., Mbaveng A. T. (2008). Antimicrobial activity of the methanolic extract and compounds from *Teclea afzelii* (Rutaceae). *South African Journal of Botany*.

[18] Dzotam J. K., Kuete V. (2017). Antibacterial and antibiotic-modifying activity of methanol extracts from six cameroonian food plants against multidrug-resistant enteric bacteria. *BioMed Research International*.

[19] Seukep J. A., Sandjo L. P., Ngadjui B. T., Kuete V. (2016). Antibacterial and antibiotic-resistance modifying activity of the extracts and compounds from *Nauclea pobeguinii* against Gram-negative multi-drug resistant phenotypes. *BMC Complementary and Alternative Medicine*.

[20] CLSI (2023). Performance standard for antimicrobial susceptibility testing. https://clsiorg/media/1469/m100s27_samplepdf.

[21] Mahfouz N., Caucci S., Achatz E. (2018). High genomic diversity of multi-drug resistant wastewater *Escherichia coli*. *Scientific Reports*.

[22] Kengne M. F., Mbaveng A. T., Kuete V. (2024). Antibiotic resistance profile of Staphylococcus aureus in cancer patients at Laquintinie hospital in Douala, littoral region, Cameroon. *BioMed Research International*.

[23] Lyonga E. E., Toukam M., Nkenfou C. (2015). Resistance pattern of enterobacteriaceae isolates from urinary tract infections to selected quinolones in Yaoundé. *Pan Afr Med J*.

[24] Daoud Z., Salem Sokhn E., Masri K., Matar G. M., Doron S. (2015). *Escherichia coli* isolated from urinary tract infections of lebanese patients between 2005 and 2012: epidemiology and profiles of resistance. *Frontiers of Medicine*.

[25] Njall C., Adiogo D., Bita A. (2013). Écologie bactérienne de l’infection nosocomiale au service de réanimation de l’hôpital Laquintinie de Douala, Cameroun. *Pan African Medical Journal*.

[26] Magill S. S., Edwards J. R., Bamberg W. (2014). Multistate point-prevalence survey of health care-associated infections. *New England Journal of Medicine*.

[27] Tofas P., Samarkos M., Piperaki E. T. (2017). *Pseudomonas aeruginosa* bacteraemia in patients with hematologic malignancies: risk factors, treatment and outcome. *Diagnostic Microbiology and Infectious Disease*.

[28] Cattaneo C., Antoniazzi F., Casari S. (2012). *P. aeruginosa* bloodstream infections among hematological patients: an old or new question?. *Annals of Hematology*.

[29] Singh R., Jain S., Chabbra R., Naithani R., Upadhyay A., Walia M. (2014). Characterization and anti-microbial susceptibility of bacterial isolates: Experience from a tertiary care cancer center in Delhi. *Indian Journal of Cancer*.

[30] Kumar V., Bhatnagar S., Gupta N. (2019). Microbial and antibiotic susceptibility profile among isolates of clinical samples of cancer patients admitted in the intensive care unit at regional tertiary care cancer center: A retrospective observational study. *Indian Journal of Critical Care Medicine*.

[31] Satlin M. J., Chavda K. D., Baker T. M. (2018). Colonization with levofloxacin-resistant extended-spectrum *β*-Lactamase-producing enterobacteriaceae and risk of bacteremia in hematopoietic stem cell transplant recipients. *Clinical Infectious Diseases*.

[32] Kim H. S., Park B. K., Kim S. K. (2017). Clinical characteristics and outcomes of *Pseudomonas aeruginosa* bacteremia in febrile neutropenic children and adolescents with the impact of antibiotic resistance: a retrospective study. *BMC Infectious Diseases*.

[33] Trecarichi E. M., Tumbarello M., Caira M. (2011). Multidrug resistant *Pseudomonas aeruginosa* bloodstream infection in adult patients with hematologic malignancies. *Haematologica*.

[34] Michel J., Jacobs J. Y., Sacks T. (1979). Bactericidal effect of combinations of antimicrobial drugs and antineoplastic antibiotics against gram-negative bacilli. *Antimicrobial Agents and Chemotherapy*.

[35] Breijyeh Z., Jubeh B., Karaman R. (2020). Resistance of gram-negative bacteria to current antibacterial agents and approaches to resolve it. *Molecules*.

[36] Moody M. R., Morris M. J., Young V. M., Moyé L. A., Schimpff S. C., Wiernik P. H. (1978). Effect of two cancer chemotherapeutic agents on the antibacterial activity of three antimicrobial agents. *Antimicrobial Agents and Chemotherapy*.

[37] Nakamura S., Oda Y., Shimada T., Oki I., Sugimoto K. (1987). SOS-inducing activity of chemical carcinogens and mutagens in Salmonella typhimurium TA1535/pSK1002: Examination with 151 chemicals. *Mutation Research Letters*.

[38] Kanafani Z. A., Mehio-Sibai A., Araj G. F., Kanaan M., Kanj S. S. (2005). Epidemiology and risk factors for extended-spectrum *β*-lactamase-producing organisms: A case control study at a tertiary care center in Lebanon. *American Journal of Infection Control*.

[39] Hobson C. A., Bonacorsi S., Hocquet D. (2020). Impact of anticancer chemotherapy on the extension of beta-lactamase spectrum: an example with KPC-type carbapenemase activity towards ceftazidime-avibactam. *Scientific Reports*.

[40] Oda Y. (1987). Induction of SOS responses in *Escherichia coli* by 5-fluorouracil. *Mutation Research*.

[41] Guðmundsdóttir J. S., Fredheim E. G. A., Koumans C. I. M. (2021). The chemotherapeutic drug methotrexate selects for antibiotic resistance. *EBioMedicine*.

[42] Abrar S., Vajeeha A., Ul-Ain N., Riaz S. (2017). Distribution of CTX-M group I and group III *β*-lactamases produced by *Escherichia coli* and klebsiella pneumoniae in Lahore, Pakistan. *Microbial Pathogenesis*.

[43] Sonda T., Kumburu H., van Zwetselaar M. (2018). Prevalence and risk factors for CTX-M gram-negative bacteria in hospitalized patients at a tertiary care hospital in Kilimanjaro, Tanzania. *European Journal of Clinical Microbiology & Infectious Diseases*.

[44] Quan J., Zhao D., Liu L. (2017). High prevalence of ESBL-producing *Escherichia coli* and *Klebsiella pneumoniae* in community-onset bloodstream infections in China. *Journal of Antimicrobial Chemotherapy*.

[45] Sid Ahmed M. A., Bansal D., Acharya A. (2016). Antimicrobial susceptibility and molecular epidemiology of extended-spectrum beta-lactamase-producing Enterobacteriaceae from intensive care units at Hamad Medical Corporation, Qatar. *Antimicrobial Resistance and Infection Control*.

[46] Hassan H., Abdalhamid B. (2014). Molecular characterization of extended-spectrum beta-lactamase producing Enterobacteriaceae in a Saudi Arabian tertiary hospital. *The Journal of Infection in Developing Countries*.

[47] Hassan M. I., Alkharsah K. R., Alzahrani A. J., Obeid O. E., Khamis A. H., Diab A. (2013). Detection of extended spectrum beta-lactamases-producing isolates and effect of AmpC overlapping. *The Journal of Infection in Developing Countries*.

[48] Bindayna K., Khanfar H. S., Senok A. C., Botta G. A. (2010). Predominance of CTX-M genotype among extended spectrum beta lactamase isolates in a tertiary hospital in Saudi Arabia. *Saudi Medical Journal*.

[49] Sheng W. H., Badal R. E., Hsueh P. R. (2013). Distribution of extended-spectrum *β*-lactamases, AmpC *β*-lactamases, and carbapenemases among enterobacteriaceae isolates causing intra-abdominal infections in the Asia-Pacific region: Results of the study for monitoring antimicrobial resistance trends (SMART). *Antimicrobial Agents and Chemotherapy*.

[50] Olowo-Okere A., Ibrahim Y. K. E., Olayinka B. O. (2018). Molecular characterisation of extended-spectrum *β*-lactamase-producing g. *Journal of Global Antimicrobial Resistance*.

[51] Kpoda D. S., Ajayi A., Somda M. (2018). Distribution of resistance genes encoding ESBLs in Enterobacteriaceae isolated from biological samples in health centers in Ouagadougou, Burkina Faso. *BMC Research Notes*.

