# Supplementary material for: Frequency of Fecal Carriage of ESBL Resistance Genes in Multidrug-Resistant Pseudomonas aeruginosa Isolates from Cancer Patients at Laquintinie Hospital, Douala, Littoral Region, Cameroon
Source: Int J Microbiol. 2024 Jun 11;2024:7685878. doi: 10.1155/2024/7685878 (PMC11222006; doi:10.1155/2024/7685878)
Supplement: Supplementary Materials — All raw data generated to support the results of this study are available in the following: S1: general information on the study population; S2: detection of Pseudomonas aeruginosa and antibiogram; and S3: detection of resistance genes. [file 7685878.f1.docx]

**S1. General information on the study population**

| Patients | Cancer | Sexe | Age | Stage of the disease | Classification | Under chemotherapy? | Pseudomonas infection |
| --- | --- | --- | --- | --- | --- | --- | --- |
| K001 | yes | M | 30 | Stage 3 | Nodes | no | no |
| K002 | yes | M | 54 | Stage 4 | metastase | no | no |
| K003 | yes | F | 50 | Stage 4 | metastase | yes | no |
| K004 | yes | F | 49 | Stage 4 | metastase | yes | no |
| K005 | yes | M | 31 | Stage 4 | metastase | yes | yes |
| K006 | yes | M | 59 | Stage 4 | metastase | no | no |
| K007 | yes | F | 67 | Stage 3 | Nodes | no | no |
| K008 | yes | M | 38 | Unknown |  | no | no |
| K009 | yes | F | 42 | Stage 3 | Nodes | yes | no |
| K010 | yes | F | 43 | Stage 4 | metastase | no | no |
| K011 | yes | F | 41 | Stage 3 | Nodes | no | no |
| K012 | yes | M | 82 | Stage 4 | metastase | yes | no |
| K013 | yes | F | 54 | Stage 4 | metastase | yes | no |
| K014 | yes | M | 18 | Stage 4 | metastase | no | no |
| K015 | yes | F | 33 | Stage 3 | Nodes | yes | no |
| K016 | yes | M | 42 | Unknown |  | no | no |
| K017 | yes | M | 68 | Stage 4 | metastase | yes | no |
| K018 | yes | M | 66 | Stage 4 | metastase | yes | no |
| K019 | yes | M | 70 | Stage 4 | metastase | no | no |
| K020 | yes | F | 43 | Stage 3 | Nodes | yes | no |
| K021 | yes | M | 61 | Stage 3 | Nodes | no | no |
| K022 | yes | M | 64 | Stage 3 | Nodes | yes | no |
| K023 | yes | M | 57 | Stage 3 | metastase | yes | no |
| K024 | yes | M | 53 | Unknown |  | no | no |
| K025 | yes | F | 64 | Unknown |  | no | no |
| K026 | yes | M | 29 | Unknown |  | yes | no |
| K027 | yes | M | 75 | Stage 3 | Nodes | no | no |
| K028 | yes | F | 62 | Stage 3 | Nodes | no | no |
| K029 | yes | F | 58 | Stage 3 | Nodes | no | no |
| K030 | yes | F | 40 | Unknown |  | no | no |
| K031 | yes | M | 65 | Stage 3 | Nodes | no | no |
| K032 | yes | F | 50 | Stage 4 | metastase | no | no |
| K033 | yes | F | 27 | Stage 4 | metastase | no | yes |
| K034 | yes | F | 38 | Stage 3 | Nodes | no | yes |
| K035 | yes | F | 43 | Stage 3 | Nodes | yes | no |
| K036 | yes | F | 52 | Stage 4 | metastase | yes | no |
| K037 | yes | F | 40 | Stage 3 | Nodes | yes | no |
| K038 | yes | M | 43 | Stage 4 | metastase | yes | no |
| K039 | yes | M | 74 | Stage 4 | metastase | no | yes |
| K040 | yes | M | 75 | Stage 4 | metastase | no | no |
| K041 | yes | M | 51 | Stage 4 | metastase | no | no |
| K042 | yes | F | 74 | Unknown |  | no | no |
| K043 | yes | F | 72 | Stage 3 | Nodes | no | no |
| K044 | yes | F | 43 | Stage 4 | metastase | no | no |
| K045 | yes | M | 56 | Stage 4 | metastase | no | yes |
| K046 | yes | F | 67 | Stage 3 | Nodes | yes | no |
| K047 | yes | M | 43 | Unknown |  | yes | no |
| K048 | yes | F | 55 | Stage 3 | Nodes | yes | no |
| K049 | yes | F | 50 | Stage 4 | metastase | no | no |
| K050 | yes | M | 50 | Stage 4 | metastase | no | no |
| K051 | yes | F | 42 | Stage 4 | metastase | no | no |
| K052 | yes | F | 45 | Stage 4 | metastase | no | no |
| K053 | yes | F | 47 | Stage 3 | Nodes | no | no |
| K054 | yes | F | 63 | Stage 4 | metastase | no | no |
| K055 | yes | F | 10 | Stage 4 | metastase | yes | no |
| K056 | yes | F | 69 | Stage 4 | metastase | yes | no |
| K057 | yes | M | 66 | Stage 4 | metastase | yes | no |
| K058 | yes | F | 59 | Stage 4 | metastase | yes | yes |
| K059 | yes | M | 60 | Stage 3 | Nodes | no | no |
| K060 | yes | F | 56 | Stage 4 | metastase | yes | yes |
| K061 | yes | F | 40 | Stage 4 | metastase | no | no |
| K062 | yes | F | 38 | Stage 3 | Nodes | no | no |
| K063 | yes | M | 78 | Stage 4 | metastase | yes | no |
| K064 | yes | F | 60 | Stage 4 | metastase | yes | no |
| K065 | yes | M | 68 | Unknown |  | no | no |
| K066 | yes | M | 77 | Stage 3 | Nodes | yes | no |
| K067 | yes | M | 39 | Stage 4 | metastase | no | no |
| K068 | yes | F | 36 | Stage 3 | Nodes | yes | no |
| K069 | yes | M | 34 | Stage 4 | metastase | no | no |
| K070 | yes | M | 29 | Stage 4 | metastase | no | no |
| K071 | yes | M | 55 | Stage 4 | metastase | no | no |
| K072 | yes | F | 57 | Stage 4 | metastase | no | no |
| K073 | yes | F | 41 | Stage 4 | metastase | no | no |
| K074 | yes | F | 23 | Stage 4 | metastase | no | no |
| K075 | yes | F | 65 | Stage 4 | metastase | yes | no |
| K076 | yes | F | 75 | Stage 4 | metastase | no | no |
| K077 | yes | M | 47 | Stage 4 | metastase | yes | no |
| K078 | yes | M | 57 | Stage 3 | Nodes | no | no |
| K079 | yes | F | 50 | Stage 4 | metastase | yes | no |
| K080 | yes | F | 41 | Unknown |  | yes | no |
| K081 | yes | F | 60 | Stage 4 | metastase | no | yes |
| K082 | yes | F | 51 | Stage 3 | Nodes | no | no |
| K083 | yes | F | 49 | Stage 3 | Nodes | yes | no |
| K084 | yes | M | 70 | Unknown |  | yes | no |
| K085 | yes | F | 44 | Stage 4 | metastase | no | no |
| K086 | yes | M | 70 | Stage 4 | metastase | no | no |
| K087 | yes | F | 67 | Stage 3 | Nodes | yes | no |
| K088 | yes | F | 44 | Stage 3 | Nodes | yes | no |
| K089 | yes | M | 75 | Stage 4 | metastase | no | no |
| K090 | yes | M | 50 | Unknown |  | no | yes |
| K091 | yes | M | 58 | Stage 3 | Nodes | no | no |
| K092 | yes | F | 37 | Stage 3 | Nodes | no | no |
| K093 | yes | F | 48 | Stage 3 | Nodes | yes | no |
| K094 | yes | M | 33 | Unknown |  | no | no |
| K095 | yes | F | 50 | Unknown |  | yes | no |
| K096 | yes | M | 69 | Stage 3 | Nodes | yes | yes |
| K097 | yes | F | 48 | Stage 3 | Nodes | yes | no |
| K098 | yes | F | 15 | Unknown |  | yes | no |
| K099 | yes | M | 58 | Stage 4 | metastase | yes | no |
| K100 | yes | F | 52 | Stage 4 | metastase | yes | no |
| K101 | yes | F | 29 | Unknown |  | yes | no |
| K102 | yes | M | 13 | Unknown |  | yes | no |
| K103 | yes | M | 32 | Stage 2 | Nodes | yes | no |
| K104 | yes | F | 54 | Stage 4 | metastase | no | no |
| K105 | yes | F | 56 | Stage 4 | metastase | yes | no |
| K106 | yes | F | 41 | Stage 4 | metastase | yes | no |
| K107 | yes | M | 11 | Unknown |  | yes | no |
| K108 | yes | F | 44 | Stage 3 | Nodes | yes | yes |
| K109 | yes | F | 43 | Stage 4 | metastase | yes | no |
| K110 | yes | F | 38 | Stage 3 | Nodes | yes | yes |
| K111 | yes | F | 12 | Unknown |  | yes | no |
| K112 | yes | F | 41 | Stage 3 | Nodes | yes | no |
| K113 | yes | M | 57 | Stage 3 | Nodes | yes | no |
| K114 | yes | F | 38 | Stage 3 | Nodes | yes | no |
| K115 | yes | F | 54 | Stage 3 | Nodes | yes | no |
| K116 | yes | F | 44 | Stage 3 | Nodes | yes | no |
| K117 | yes | F | 50 | Unknown |  | yes | no |
| K118 | yes | M | 71 | Stage 4 | metastase | yes | yes |
| K119 | yes | F | 68 | Stage 3 | Nodes | yes | no |
| K120 | yes | F | 61 | Stage 4 | metastase | yes | no |
| K121 | yes | F | 60 | Stage 3 | Nodes | yes | no |
| K122 | yes | F | 49 | Unknown |  | yes | no |
| K123 | yes | F | 39 | Stage 3 | Nodes | yes | no |
| K124 | yes | M | 37 | Unknown |  | yes | no |
| K125 | yes | F | 64 | Stage 3 | Nodes | yes | no |
| K126 | yes | F | 36 | Stage 4 | metastase | yes | yes |
| K127 | yes | F | 45 | Unknown |  | yes | no |
| K128 | yes | F | 50 | Stage 3 | Nodes | yes | no |
| K129 | yes | F | 30 | Stage 3 | Nodes | yes | no |
| K130 | yes | F | 45 | Stage 4 | metastase | no | yes |
| K131 | yes | F | 47 | Stage 4 | metastase | no | yes |
| K132 | yes | F | 76 | Stage 4 | metastase | no | yes |
| K133 | yes | F | 40 | Stage 3 | Nodes | no | no |
| K134 | yes | F | 27 | Stage 4 | metastase | no | no |
| K135 | yes | M | 80 | Stage 4 | metastase | no | no |
| K136 | yes | F | 55 | Stage 4 | metastase | no | yes |
| K137 | yes | M | 11 | Stage 4 | metastase | yes | no |
| K138 | yes | F | 50 | Stage 3 | Nodes | yes | yes |
| K139 | yes | M | 10 | Stage 4 | metastase | yes | yes |
| K140 | yes | M | 63 | Stage 4 | metastase | yes | no |
| K141 | yes | F | 19 | Stage 3 | Nodes | yes | no |
| K142 | yes | M | 42 | Stage 4 | metastase | yes | yes |
| K143 | yes | M | 32 | Stage 4 | metastase | yes | no |
| K144 | yes | M | 46 | Unknown |  | yes | no |
| K145 | yes | F | 54 | Stage 4 | metastase | yes | yes |
| K146 | yes | F | 43 | Stage 4 | metastase | no | no |
| K147 | yes | M | 58 | Stage 4 | metastase | yes | yes |
| K148 | yes | F | 72 | Unknown |  | yes | no |
| K149 | yes | F | 49 | Stage 4 | metastase | yes | no |
| K150 | yes | F | 79 | Stage 4 | metastase | yes | no |
| K151 | yes | F | 62 | Stage 4 | metastase | no | yes |
| K152 | yes | F | 66 | Stage 4 | metastase | yes | no |
| K153 | yes | F | 66 | Stage 3 | Nodes | no | no |
| K154 | yes | F | 51 | Stage 4 | metastase | yes | no |
| K155 | yes | F | 46 | Stage 4 | metastase | yes | no |
| K156 | yes | F | 60 | Stage 4 | metastase | yes | no |
| K157 | yes | F | 68 | Stage 4 | metastase | yes | no |
| K158 | yes | M | 61 | Stage 4 | metastase | yes | no |
| K159 | yes | M | 45 | Unknown |  | yes | yes |
| K160 | yes | F | 47 | Stage 4 | metastase | yes | no |
| K161 | yes | F | 32 | Unknown |  | yes | no |
| K162 | yes | F | 54 | Stage 4 | metastase | yes | no |
| K163 | yes | F | 64 | Stage 3 | Nodes | yes | no |
| K164 | yes | F | 48 | Stage 4 | metastase | yes | no |
| K165 | yes | M | 43 | Stage 3 | Nodes | yes | no |
| K166 | yes | M | 64 | Stage 4 | metastase | yes | no |
| K167 | yes | F | 60 | Stage 4 | metastase | no | no |
| K168 | yes | M | 56 | Unknown |  | yes | no |
| K169 | yes | M | 47 | Stage 4 | metastase | yes | no |
| K170 | yes | F | 56 | Stage 4 | metastase | yes | no |
| K171 | yes | F | 39 | Stage 4 | metastase | no | no |
| K172 | yes | F | 31 | Stage 4 | metastase | no | yes |
| K173 | yes | M | 57 | Stage 3 | metastase | yes | no |
| K174 | yes | M | 54 | Stage 4 | metastase | yes | no |
| K175 | yes | M | 28 | Stage 4 | metastase | no | no |
| K176 | yes | M | 45 | Stage 4 | metastase | no | yes |
| K177 | yes | M | 62 | Stage 4 | metastase | no | no |
| K178 | yes | M | 62 | Stage 3 | Nodes | yes | no |
| K179 | yes | M | 55 | Stage 3 | Nodes | yes | no |
| K180 | yes | F | 46 | Stage 3 | Nodes | yes | no |
| K181 | yes | M | 42 | Unknown |  | yes | no |
| K182 | yes | M | 72 | Unknown |  | yes | no |
| K183 | yes | F | 45 | Stage 2 | Tumeur | no | no |
| K184 | yes | M | 69 | Stage 4 | metastase | yes | no |
| K185 | yes | F | 36 | Unknown |  | yes | no |
| K186 | yes | M | 41 | Stage 3 | Nodes | yes | no |
| K187 | yes | F | 47 | Stage 4 | metastase | yes | yes |
| K188 | yes | M | 67 | Stage 4 | metastase | no | yes |
| K189 | yes | F | 63 | Stage 4 | metastase | no | no |
| K190 | yes | F | 66 | Stage 4 | metastase | no | no |
| K191 | yes | M | 28 | Unknown |  | yes | no |
| K192 | yes | M | 54 | Unknown |  | yes | no |
| K193 | yes | M | 41 | Stage 4 | metastase | no | no |
| K194 | yes | F | 36 | Stage 4 | metastase | yes | no |
| K195 | yes | M | 48 | Stage 4 | metastase | yes | no |
| K196 | yes | F | 64 | Stage 4 | metastase | yes | yes |
| K197 | yes | M | 60 | Stage 4 | metastase | yes | yes |
| K198 | yes | M | 45 | Stage 4 | metastase | yes | no |
| K199 | yes | M | 41 | Stage 4 | metastase | no | no |
| K200 | yes | F | 54 | Stage 4 | metastase | yes | no |
| K201 | yes | M | 37 | Stage 4 | metastase | yes | no |
| K202 | yes | M | 39 | Stage 4 | metastase | no | no |
| K203 | yes | M | 43 | Stage 3 | Nodes | no | no |
| K204 | yes | F | 60 | Stage 4 | metastase | yes | no |
| K205 | yes | F | 43 | Stage 4 | metastase | no | yes |
| K206 | yes | F | 40 | Stage 3 | Nodes | yes | no |
| K207 | yes | M | 44 | Stage 3 | Nodes | yes | no |
| K208 | yes | F | 61 | Stage 4 | metastase | yes | no |
| K209 | yes | F | 56 | Stage 4 | metastase | yes | yes |
| K210 | yes | F | 31 | Stage 3 | Nodes | no | yes |
| K211 | yes | F | 62 | Stage 3 | Nodes | yes | no |
| K212 | yes | M | 52 | Stage 3 | Nodes | yes | no |
| K213 | yes | F | 63 | Stage 4 | metastase | yes | no |
| K214 | yes | F | 39 | Unknown |  | yes | no |
| K215 | yes | F | 45 | Stage 4 | metastase | yes | no |
| K216 | yes | M | 44 | Unknown |  | yes | yes |
| K217 | yes | F | 31 | Stage 4 | metastase | yes | no |
| K218 | yes | F | 47 | Stage 4 | metastase | yes | no |
| K219 | yes | F | 36 | Stage 3 | Nodes | yes | yes |
| K220 | yes | F | 28 | Stage 4 | metastase | no | no |
| K221 | yes | F | 47 | Stage 4 | metastase | no | no |
| K222 | yes | F | 42 | Stage 4 | metastase | yes | no |
| K223 | yes | F | 72 | Stage 3 | Nodes | no | no |
| K224 | yes | F | 49 | Stage 4 | metastase | no | no |
| K225 | yes | F | 36 | Unknown |  | yes | yes |
| K226 | yes | F | 45 | Stage 3 | Nodes | yes | no |
| K227 | yes | F | 75 | Stage 4 | metastase | no | no |
| K228 | yes | F | 29 | Stage 4 | metastase | no | no |
| K229 | yes | M | 46 | Unknown |  | yes | no |
| K230 | yes | F | 45 | Stage 3 | Nodes | yes | no |
| K231 | yes | F | 54 | Stage 4 | metastase | yes | no |
| K232 | yes | F | 47 | Stage 4 | metastase | yes | no |
| K233 | yes | F | 49 | Stage 4 | metastase | no | no |
| K234 | yes | F | 36 | Stage 4 | metastase | no | yes |
| K235 | yes | F | 40 | Stage 4 | metastase | no | no |
| K236 | yes | M | 81 | Stage 4 | metastase | no | no |
| K237 | yes | F | 61 | Stage 4 | metastase | no | no |
| K238 | yes | F | 68 | Stage 3 | Nodes | no | no |
| K239 | yes | F | 20 | Stage 4 | metastase | no | no |
| K240 | yes | M | 80 | Stage 4 | metastase | no | no |
| K241 | yes | F | 56 | Stage 4 | metastase | yes | no |
| K242 | yes | F | 39 | Stage 3 | Nodes | yes | no |
| K243 | yes | F | 45 | Stage 4 | metastase | yes | no |
| K244 | yes | F | 38 | Stage 3 | Nodes | yes | no |
| K245 | yes | F | 33 | Stage 4 | metastase | yes | no |
| K246 | yes | F | 76 | Stage 4 | metastase | yes | no |
| K247 | yes | F | 62 | Stage 4 | metastase | no | no |
| K248 | yes | F | 44 | Stage 4 | metastase | no | no |
| K249 | yes | F | 43 | Unknown |  | yes | no |
| K250 | yes | F | 50 | Stage 4 | metastase | no | no |
| K251 | yes | F | 45 | Stage 4 | metastase | yes | no |
| K252 | yes | M | 14 | Stage 4 | metastase | yes | no |
| K253 | yes | F | 43 | Stage 4 | metastase | no | no |
| K254 | yes | F | 61 | Stage 4 | metastase | yes | no |
| K255 | yes | M | 71 | Stage 4 | metastase | yes | no |
| K256 | yes | M | 30 | Stage 4 | metastase | no | no |
| K257 | yes | M | 61 | Stage 4 | metastase | no | no |
| K258 | yes | F | 44 | Stage 4 | metastase | no | no |
| K259 | yes | F | 62 | Stage 4 | metastase | yes | no |
| K260 | yes | F | 46 | Stage 3 | Nodes | yes | no |
| K261 | yes | F | 47 | Unknown |  | yes | yes |
| K262 | yes | M | 47 | Stage 3 | Nodes | yes | no |
| K263 | yes | F | 35 | Stage 3 | Nodes | yes | no |
| K264 | yes | M | 45 | Unknown |  | no | yes |
| K265 | yes | F | 43 | Stage 3 | Nodes | yes | no |
| K266 | yes | F | 46 | Stage 4 | metastase | yes | no |
| K267 | yes | F | 54 | Unknown |  | yes | no |
| K268 | yes | F | 69 | Stage 4 | metastase | yes | no |
| K269 | yes | F | 71 | Stage 4 | metastase | yes | no |
| K270 | yes | F | 69 | Stage 4 | metastase | yes | no |
| K271 | yes | F | 58 | Stage 3 | Nodes | yes | no |
| K272 | yes | F | 46 | Stage 4 | metastase | no | yes |
| K273 | yes | F | 52 | Stage 4 | metastase | no | no |
| K274 | yes | F | 39 | Stage 4 | metastase | yes | no |
| K275 | yes | F | 52 | Stage 4 | metastase | yes | no |
| K276 | yes | F | 48 | Stage 4 | metastase | yes | no |
| K277 | yes | F | 53 | Stage 4 | metastase | yes | no |
| K278 | yes | F | 58 | Stage 3 | Nodes | yes | no |
| K279 | yes | F | 45 | Stage 4 | metastase | yes | no |
| K280 | yes | M | 41 | Stage 4 | metastase | no | no |
| K281 | yes | F | 49 | Stage 3 | Nodes | yes | no |
| K282 | yes | F | 53 | Stage 4 | metastase | no | yes |
| K283 | yes | F | 60 | Stage 4 | metastase | no | no |
| K284 | yes | F | 46 | Stage 4 | metastase | no | yes |
| K285 | yes | F | 57 | Stage 4 | metastase | no | no |
| K286 | yes | M | 47 | Stage 4 | metastase | no | no |
| K287 | yes | M | 56 | Stage 4 | metastase | yes | no |
| K288 | yes | F | 26 | Unknown |  | yes | no |
| K289 | yes | M | 28 | Stage 3 | Nodes | yes | no |
| K290 | yes | F | 60 | Stage 4 | metastase | yes | yes |
| K291 | yes | F | 32 | Stage 3 | Nodes | yes | no |
| K292 | yes | M | 27 | Stage 4 | metastase | yes | no |
| K293 | yes | M | 42 | Stage 4 | metastase | no | no |
| K294 | yes | F | 41 | Stage 4 | metastase | yes | yes |
| K295 | yes | M | 28 | Stage 3 | Nodes | yes | no |
| K296 | yes | F | 48 | Stage 3 | Nodes | yes |  |
| K297 | yes | F | 68 | Stage 4 | metastase | yes | no |
| K298 | yes | F | 64 | Unknown |  | yes |  |
| K299 | yes | M | 59 | Stage 4 | metastase | no | no |
| K300 | yes | M | 52 | Stage 4 | metastase | no |  |
| K301 | yes | M | 25 | Stage 4 | metastase | no | no |
| K302 | yes | F | 75 | Stage 4 | metastase | no |  |
| K303 | yes | F | 49 | Stage 4 | metastase | no | no |
| K304 | yes | F | 41 | Stage 4 | metastase | yes |  |
| K305 | yes | F | 63 | Stage 4 | metastase | yes | no |
| K306 | yes | M | 16 | Stage 4 | metastase | yes |  |
| K307 | yes | F | 50 | Stage 4 | metastase | yes | no |
|  |  |  |  |  |  |  |  |
|  |  |  |  |  |  |  | no |
| D001 | no | M | 45 |  |  |  |  |
| D002 | no | F | 64 |  |  |  | no |
| D003 | no | F | 52 |  |  |  |  |
| D004 | no | M | 42 |  |  |  | no |
| D005 | no | M | 69 |  |  |  |  |
| D006 | no | F | 34 |  |  |  | no |
| D007 | no | M | 49 |  |  |  |  |
| D008 | no | F | 44 |  |  |  | no |
| D009 | no | M | 77 |  |  |  |  |
| D010 | no | M | 10 |  |  |  | no |
| D011 | no | M | 40 |  |  |  |  |
| D012 | no | M | 31 |  |  |  | no |
| D013 | no | F | 38 |  |  |  | yes |
| D014 | no | F | 55 |  |  |  | no |
| D015 | no | F | 41 |  |  |  | no |
| D016 | no | F | 20 |  |  |  | no |
| D017 | no | F | 61 |  |  |  | no |
| D018 | no | F | 23 |  |  |  | no |
| D019 | no | F | 53 |  |  |  | no |
| D020 | no | F | 34 |  |  |  | no |
| D021 | no | F | 36 |  |  |  | no |
| D022 | no | M | 10 |  |  |  | no |
| D023 | no | M | 28 |  |  |  | no |
| D024 | no | F | 22 |  |  |  | no |
| D025 | no | F | 38 |  |  |  | no |
| D026 | no | F | 20 |  |  |  | no |
| D027 | no | M | 35 |  |  |  | yes |
| D028 | no | F | 40 |  |  |  | no |
| D029 | no | F | 53 |  |  |  | yes |
| D030 | no | F | 45 |  |  |  | no |
| D031 | no | F | 36 |  |  |  | no |
| D032 | no | M | 63 |  |  |  | no |
| D033 | no | F | 22 |  |  |  | yes |
| D034 | no | F | 38 |  |  |  | no |
| D035 | no | M | 57 |  |  |  | no |
| D036 | no | F | 20 |  |  |  | no |
| D037 | no | F | 54 |  |  |  | no |
| D038 | no | F | 27 |  |  |  | no |
| D039 | no | F | 25 |  |  |  | no |
| D040 | no | F | 58 |  |  |  | no |
| D041 | no | M | 27 |  |  |  | no |
| D042 | no | F | 66 |  |  |  | no |
| D043 | no | M | 10 |  |  |  | no |
| D044 | no | F | 37 |  |  |  | yes |
| D045 | no | F | 78 |  |  |  | no |
| D046 | no | M | 63 |  |  |  | yes |
| D047 | no | M | 43 |  |  |  | no |
| D048 | no | F | 63 |  |  |  | no |
| D049 | no | M | 52 |  |  |  | yes |
| D050 | no | F | 23 |  |  |  | no |
| D051 | no | M | 42 |  |  |  | no |
| D052 | no | F | 23 |  |  |  | no |
| D053 | no | F | 54 |  |  |  | yes |
| D054 | no | F | 31 |  |  |  | no |
| D055 | no | M | 44 |  |  |  | no |
| D056 | no | M | 43 |  |  |  | no |
| D057 | no | M | 40 |  |  |  | no |
| D058 | no | M | 47 |  |  |  | no |
| D059 | no | M | 10 |  |  |  | no |
| D060 | no | M | 41 |  |  |  | no |
| D061 | no | M | 37 |  |  |  | no |
| D062 | no | F | 17 |  |  |  | no |
| D063 | no | F | 30 |  |  |  | no |
| D064 | no | F | 38 |  |  |  | no |
| D065 | no | M | 30 |  |  |  | no |
| D066 | no | F | 33 |  |  |  | no |
| D067 | no | F | 84 |  |  |  | no |
| D068 | no | F | 38 |  |  |  | no |
| D069 | no | M | 48 |  |  |  | yes |
| D070 | no | F | 39 |  |  |  | no |
| D071 | no | F | 44 |  |  |  | no |
| D072 | no | F | 34 |  |  |  | no |
| D073 | no | F | 10 |  |  |  | no |
| D074 | no | F | 42 |  |  |  | yes |
| D075 | no | F | 36 |  |  |  | no |
| D076 | no | M | 29 |  |  |  | no |
| D077 | no | F | 20 |  |  |  | no |
| D078 | no | M | 29 |  |  |  | no |
| D079 | no | F | 10 |  |  |  | yes |
| D080 | no | M | 81 |  |  |  | no |
| D081 | no | F | 29 |  |  |  | no |
| D082 | no | F | 51 |  |  |  | no |
| D083 | no | M | 35 |  |  |  | yes |
| D084 | no | F | 51 |  |  |  | no |
| D085 | no | F | 42 |  |  |  | no |
| D086 | no | M | 64 |  |  |  | no |
| D087 | no | M | 56 |  |  |  | no |
| D088 | no | F | 33 |  |  |  | no |
| D089 | no | M | 34 |  |  |  | no |
| D090 | no | M | 58 |  |  |  | no |
| D091 | no | M | 54 |  |  |  | no |
| D092 | no | M | 49 |  |  |  | no |
| D093 | no | M | 48 |  |  |  | no |
| D094 | no | M | 52 |  |  |  | no |
| D095 | no | F | 34 |  |  |  | no |
| D096 | no | F | 29 |  |  |  | no |
| D097 | no | M | 25 |  |  |  | no |
| D098 | no | F | 55 |  |  |  | no |
| D099 | no | M | 41 |  |  |  | no |
| D100 | no | F | 48 |  |  |  | yes |
| D101 | no | F | 42 |  |  |  | no |
| D102 | no | F | 45 |  |  |  | no |
| D103 | no | F | 23 |  |  |  | no |
| D104 | no | M | 47 |  |  |  | no |
| D105 | no | M | 15 |  |  |  | no |
| D106 | no | F | 68 |  |  |  | no |
| D107 | no | M | 39 |  |  |  | no |
| D108 | no | M | 47 |  |  |  | no |
| D109 | no | F | 36 |  |  |  | no |
| D110 | no | M | 45 |  |  |  | no |
| D111 | no | F | 42 |  |  |  | no |
| D112 | no | F | 20 |  |  |  | no |
| D113 | no | F | 23 |  |  |  | no |
| D114 | no | M | 47 |  |  |  | no |
| D115 | no | M | 20 |  |  |  | no |
| D116 | no | F | 68 |  |  |  | no |
| D117 | no | M | 39 |  |  |  | no |
| D118 | no | M | 47 |  |  |  | no |
| D119 | no | F | 36 |  |  |  | no |
| D120 | no | M | 32 |  |  |  | no |
| D121 | no | F | 80 |  |  |  | no |
| D122 | no | F | 53 |  |  |  | no |
| D123 | no | F | 29 |  |  |  | no |
| D124 | no | M | 66 |  |  |  | no |
| D125 | no | M | 71 |  |  |  | no |
| D126 | no | M | 42 |  |  |  | no |
| D127 | no | F | 28 |  |  |  | no |
| D128 | no | M | 33 |  |  |  | no |
| D129 | no | F | 72 |  |  |  | no |
| D130 | no | F | 60 |  |  |  | no |
| D131 | no | F | 70 |  |  |  | no |
| D132 | no | M | 29 |  |  |  | no |
| D133 | no | F | 35 |  |  |  | no |
| D134 | no | M | 46 |  |  |  | no |
| D135 | no | M | 39 |  |  |  | no |
| D136 | no | F | 65 |  |  |  | no |
| D137 | no | M | 44 |  |  |  | no |
| D138 | no | F | 65 |  |  |  | no |
| D139 | no | F | 44 |  |  |  | no |
| D140 | no | F | 64 |  |  |  | no |
| D141 | no | F | 56 |  |  |  | no |
| D142 | no | F | 66 |  |  |  | no |
| D143 | no | M | 34 |  |  |  | no |
| D144 | no | F | 58 |  |  |  | no |
| D145 | no | F | 54 |  |  |  | no |
| D146 | no | F | 48 |  |  |  | no |
| D147 | no | M | 52 |  |  |  | no |
| D148 | no | M | 34 |  |  |  | no |
| D149 | no | F | 29 |  |  |  | no |
| D150 | no | F | 38 |  |  |  | no |
| D151 | no | F | 36 |  |  |  | no |
| D152 | no | M | 10 |  |  |  | no |
| D153 | no | M | 28 |  |  |  | no |
| D154 | no | F | 22 |  |  |  | no |
| D155 | no | F | 38 |  |  |  | no |
| D156 | no | F | 20 |  |  |  | no |
| D157 | no | M | 35 |  |  |  | no |
| D158 | no | F | 40 |  |  |  | no |
| D159 | no | F | 53 |  |  |  | no |
| D160 | no | F | 45 |  |  |  | no |
| D161 | no | F | 36 |  |  |  | no |
| D162 | no | M | 63 |  |  |  | no |
| D163 | no | F | 22 |  |  |  | no |
| D164 | no | F | 38 |  |  |  | no |
| D165 | no | M | 57 |  |  |  | no |
| D166 | no | F | 20 |  |  |  | no |
| D167 | no | F | 54 |  |  |  | no |
| D168 | no | F | 27 |  |  |  | no |
| D169 | no | F | 25 |  |  |  | no |
| D170 | no | F | 58 |  |  |  | no |
| D171 | no | M | 27 |  |  |  | no |
| D172 | no | F | 66 |  |  |  | no |
| D173 | no | M | 10 |  |  |  | no |
| D174 | no | F | 37 |  |  |  | no |
| D175 | no | F | 78 |  |  |  | no |
| D176 | no | M | 63 |  |  |  | no |
| D177 | no | M | 43 |  |  |  | no |
| D178 | no | F | 63 |  |  |  | no |
| D179 | no | M | 52 |  |  |  | no |
| D180 | no | F | 23 |  |  |  | no |
| D181 | no | M | 42 |  |  |  | no |
| D182 | no | F | 23 |  |  |  | no |
| D183 | no | F | 54 |  |  |  | no |
| D184 | no | F | 31 |  |  |  | no |
| D185 | no | M | 44 |  |  |  | no |
| D186 | no | M | 43 |  |  |  | no |
| D187 | no | M | 40 |  |  |  | no |
| D188 | no | M | 47 |  |  |  | no |
| D189 | no | M | 10 |  |  |  | no |
| D190 | no | M | 41 |  |  |  | no |
| D191 | no | M | 37 |  |  |  | no |
| D192 | no | F | 17 |  |  |  | no |
| D193 | no | F | 30 |  |  |  | no |
| D194 | no | F | 38 |  |  |  | no |
| D195 | no | M | 30 |  |  |  | no |
| D196 | no | F | 33 |  |  |  | no |
| D197 | no | F | 84 |  |  |  | no |
| D198 | no | F | 38 |  |  |  | no |
| D199 | no | M | 48 |  |  |  | no |
| D200 | no | F | 39 |  |  |  | no |
|  | Legend: F: female; M: male | | | | | |  |
|  |  |  |  |  |  |  |  |

**S2. Detection of *Pseudomonas aeruginosa* and antibiogram**

| Patients | Cancer | IMP_PSE | AMX_PSE | CAZ_PSE | FOX_PSE | CTX_PSE | CXM_PSE | COT_PSE | AMK_PSE |  | GEN_PSE | CIP_PSE | OFX_PSE | NAL_PSE | COL_PSE | PRL_PSE | PPT_PSE | TCC_PSE | TET_PSE | NIT_PSE | AMC_PSE | RESISTANCE phenotype |
| --- | --- | --- | --- | --- | --- | --- | --- | --- | --- | --- | --- | --- | --- | --- | --- | --- | --- | --- | --- | --- | --- | --- |
| K005 | yes | R | R | R | R | R | R | R | S |  | S | R | R | R | R | R | R | R | R | R | R | MULTI RESISTANT |
| K033 | yes | R | R | R | R | R | R | R | S |  | S | R | R | R | R | R | R | R | R | R | R | MULTI RESISTANT |
| K034 | yes | R | R | R | S | R | R | S | S |  | S | R | R | R | R | R | R | R | R | S | R | MULTI RESISTANT |
| K039 | yes | R | R | R | R | R | R | R | S |  | S | R | R | R | R | R | R | R | R | R | R | MULTI RESISTANT |
| K045 | yes | R | R | R | S | R | R | R | S |  | S | R | R | R | R | R | R | R | R | S | R | MULTI RESISTANT |
| K058 | yes | R | R | R | R | R | R | R | S |  | S | R | R | R | R | R | R | R | R | R | R | MULTI RESISTANT |
| K060 | yes | R | R | R | R | R | R | R | S |  | S | R | R | R | R | R | R | R | R | R | R | MULTI RESISTANT |
| K081 | yes | R | R | R | R | R | R | R | S |  | S | R | R | R | R | R | R | R | R | R | R | MULTI RESISTANT |
| K090 | yes | R | R | R | R | R | R | R | S |  | S | R | R | R | R | R | R | R | R | R | R | MULTI RESISTANT |
| K096 | yes | R | R | R | R | R | R | R | S |  | S | R | R | R | R | R | R | R | R | R | R | MULTI RESISTANT |
| K108 | yes | R | R | R | R | R | R | R | S |  | S | R | R | R | R | R | R | R | R | R | R | MULTI RESISTANT |
| K110 | yes | R | R | R | R | R | R | R | R |  | R | R | R | R | R | R | R | R | R | R | R | MULTI RESISTANT |
| K118 | yes | R | R | R | R | R | R | R | S |  | S | R | R | R | R | R | R | R | R | R | R | MULTI RESISTANT |
| K126 | yes | R | R | R | R | R | R | R | R |  | S | R | R | R | R | R | R | R | R | S | R | MULTI RESISTANT |
| K130 | yes | R | R | R | R | R | R | R | S |  | S | R | R | R | R | R | R | R | R | R | R | MULTI RESISTANT |
| K131 | yes | R | R | R | R | R | R | R | S |  | S | R | R | R | R | R | R | R | R | R | R | MULTI RESISTANT |
| K132 | yes | R | R | R | R | R | R | R | S |  | R | R | R | R | R | R | R | R | R | R | R | MULTI RESISTANT |
| K136 | yes | R | R | R | R | R | R | R | S |  | S | R | R | R | R | R | R | R | R | R | R | MULTI RESISTANT |
| K138 | yes | R | R | R | R | R | R | R | S |  | S | R | R | R | R | R | R | R | R | R | S | MULTI RESISTANT |
| K139 | yes | I | R | R | R | R | R | R | S |  | S | R | R | R | R | R | R | R | R | R | R | MULTI RESISTANT |
| K142 | yes | R | R | R | R | R | R | R | S |  | S | R | R | R | R | R | R | R | R | R | R | MULTI RESISTANT |
| K145 | yes | R | R | R | R | R | R | R | S |  | S | R | R | R | R | R | R | R | R | R | R | MULTI RESISTANT |
| K147 | yes | R | S | R | R | R | R | R | S |  | S | R | R | R | R | R | R | R | S | R | R | MULTI RESISTANT |
| K151 | yes | I | R | R | R | R | R | R | S |  | S | R | R | R | R | R | R | R | R | R | R | MULTI RESISTANT |
| K159 | yes | I | R | R | R | R | R | R | S |  | S | S | S | R | R | R | R | R | R | R | R | MULTI RESISTANT |
| K172 | yes | R | R | R | R | R | R | R | S |  | S | R | R | R | R | R | R | R | R | R | R | MULTI RESISTANT |
| K176 | yes | R | R | R | R | R | R | R | S |  | S | R | R | R | R | R | R | R | R | R | R | MULTI RESISTANT |
| K187 | yes | R | R | R | R | R | R | R | S |  | S | R | R | R | R | R | R | R | R | R | R | MULTI RESISTANT |
| K188 | yes | R | R | R | R | R | R | R | S |  | S | R | R | R | R | R | R | R | R | R | R | MULTI RESISTANT |
| K196 | yes | R | R | R | R | R | R | R | S |  | S | R | R | R | R | R | R | R | R | R | R | MULTI RESISTANT |
| K197 | yes | R | R | R | R | S | R | S | S |  | S | R | R | R | R | R | R | R | R | R | R | MULTI RESISTANT |
| K205 | yes | R | R | R | R | R | R | R | S |  | S | R | R | R | R | R | R | R | R | R | R | MULTI RESISTANT |
| K209 | yes | R | R | R | R | R | S | R | S |  | S | R | R | S | R | R | R | R | R | R | R | MULTI RESISTANT |
| K210 | yes | R | R | R | R | R | R | R | R |  | R | R | R | R | S | R | R | R | R | R | R | MULTI RESISTANT |
| K216 | yes | R | R | R | R | R | R | R | S |  | S | R | R | R | R | R | R | R | R | R | R | MULTI RESISTANT |
| K219 | yes | R | R | R | R | R | R | R | S |  | S | R | R | R | R | R | R | R | R | R | R | MULTI RESISTANT |
| K225 | yes | R | R | R | R | R | R | R | S |  | S | R | R | R | R | R | R | R | R | R | R | MULTI RESISTANT |
| K234 | yes | R | R | R | R | R | R | R | S |  | S | R | R | R | R | R | R | R | R | R | R | MULTI RESISTANT |
| K261 | yes | I | R | R | R | R | R | R | S |  | S | R | R | R | R | R | R | R | R | R | R | MULTI RESISTANT |
| K264 | yes | I | R | R | R | R | R | R | S |  | S | R | R | R | R | R | R | R | R | R | R | MULTI RESISTANT |
| K272 | yes | R | R | R | R | R | R | R | S |  | S | R | R | R | R | R | R | R | R | R | R | MULTI RESISTANT |
| K282 | yes | I | R | R | R | R | R | R | S |  | R | R | R | R | R | R | R | R | R | R | R | MULTI RESISTANT |
| K284 | yes | S | R | R | R | R | R | R | S |  | S | I | I | R | R | R | R | R | R | R | R | MULTI RESISTANT |
| K290 | yes | S | R | R | R | R | R | R | S |  | S | S | S | R | R | R | R | R | R | R | R | MULTI RESISTANT |
| K294 | yes | R | R | R | R | R | R | R | S |  | S | R | R | R | R | R | R | R | R | R | R | MULTI RESISTANT |
| D013 | no | R | R | R | R | R | R | R | R |  | S | I | I | I | R | R | R | R | R | R | R | MULTI RESISTANT |
| D027 | no | I | R | R | R | R | I | R | S |  | S | I | I | I | R | R | R | R | R | R | R | MULTI RESISTANT |
| D029 | no | R | R | R | I | I | R | R | R |  | R | I | I | I | I | I | I | I | R | R | R | MULTI RESISTANT |
| D033 | no | I | R | R | R | R | R | R | R |  | R | R | R | R | R | R | R | R | R | R | R | MULTI RESISTANT |
| D044 | no | R | R | R | R | R | R | R | R |  | R | S | S | S | R | R | R | R | R | R | R | MULTI RESISTANT |
| D046 | no | I | R | R | I | I | R | R | R |  | R | I | I | I | R | R | R | R | R | R | R | MULTI RESISTANT |
| D049 | no | I | R | I | R | R | R | R | S |  | S | I | I | I | R | R | R | R | R | I | R | MULTI RESISTANT |
| D053 | no | I | I | I | I | R | R | R | R |  | R | I | I | I | R | R | I | I | I | R | R | MULTI RESISTANT |
| D069 | no | I | R | I | R | R | R | R | R |  | R | I | I | I | R | R | R | R | R | R | R | RESISTANCE |
| D074 | no | R | R | R | R | R | R | R | S |  | S | I | I | I | R | R | R | R | R | R | R | MULTI RESISTANT |
| D079 | no | I | R | R | R | R | R | R | R |  | R | I | I | I | R | R | R | R | R | R | R | MULTI RESISTANT |
| D083 | no | I | R | R | R | R | R | R | S |  | S | I | I | I | R | R | S | R | R | R | R | MULTI RESISTANT |
| D100 | no | I | I | I | I | R | R | R | R |  | R | I | I | I | R | R | I | R | R | R | R | RESISTANCE |
|  |  |  |  |  |  |  |  |  |  |  |  |  |  |  |  |  |  |  |  |  |  |  |
|  |  |  |  |  |  |  |  |  |  |  |  |  |  |  |  |  |  |  |  |  |  |  |

**S3. Detection of resistance genes**

| Patients | Cancer | BLA TEM | BLA OXA | BLA SHV | BLA CTX-M | BlaTEM+Bla CTX-M | BlaTEM+BlaOXA | BlaTEM+BlaSHV | Bla CTX-M+BlaOXA | Bla CTX-M+BlaSHV |
| --- | --- | --- | --- | --- | --- | --- | --- | --- | --- | --- |
| K005 | yes | yes | no | yes | yes | yes | no | yes | no | yes |
| K033 | yes | yes | yes | yes | yes | yes | yes | yes | yes | yes |
| K034 | yes | yes | yes | no | yes | yes | yes | no | yes | no |
| K039 | yes | yes | yes | yes | yes | yes | yes | yes | yes | yes |
| K045 | yes | yes | yes | yes | yes | yes | yes | yes | yes | yes |
| K058 | yes | yes | yes | no | yes | yes | yes | no | yes | no |
| K060 | yes | yes | yes | no | yes | yes | yes | no | yes | no |
| K081 | yes | yes | no | no | yes | yes | no | no | no | no |
| K090 | yes | yes | no | yes | yes | yes | no | yes | no | yes |
| K096 | yes | no | yes | yes | yes | no | no | no | yes | yes |
| K108 | yes | yes | yes | yes | yes | yes | yes | yes | yes | yes |
| K110 | yes | yes | no | no | yes | yes | no | no | no | no |
| K118 | yes | yes | yes | yes | yes | yes | yes | yes | yes | yes |
| K126 | yes | yes | no | yes | yes | yes | no | yes | no | yes |
| K130 | yes | yes | no | yes | yes | yes | no | yes | no | yes |
| K131 | yes | no | yes | no | no | no | no | no | no | no |
| K132 | yes | yes | no | no | no | no | no | no | no | no |
| K136 | yes | no | no | yes | yes | no | no | no | no | yes |
| K138 | yes | yes | yes | no | yes | yes | yes | no | yes | no |
| K139 | yes | yes | yes | yes | yes | yes | yes | yes | yes | yes |
| K142 | yes | yes | yes | no | yes | yes | yes | no | yes | no |
| K145 | yes | yes | yes | yes | yes | yes | yes | yes | yes | yes |
| K147 | yes | yes | yes | yes | yes | yes | yes | yes | yes | yes |
| K151 | yes | yes | yes | yes | yes | yes | yes | yes | yes | yes |
| K159 | yes | yes | yes | yes | yes | yes | yes | yes | yes | yes |
| K172 | yes | no | no | no | yes | no | no | no | no | no |
| K176 | yes | yes | yes | no | no | no | no | no | no | no |
| K187 | yes | yes | no | no | no | no | no | no | no | no |
| K188 | yes | yes | yes | no | yes | yes | yes | no | yes | no |
| K196 | yes | yes | no | yes | yes | yes | no | yes | no | yes |
| K197 | yes | yes | no | yes | no | no | no | yes | no | no |
| K205 | yes | yes | no | no | no | no | no | no | no |  |
| K209 | yes | yes | no | no | yes | yes | no | no | no | no |
| K210 | yes | no | no | no | yes | no | no | no | no | no |
| K216 | yes | no | yes | no | yes | no | no | no | yes | no |
| K219 | yes | yes | no | yes | yes | yes | no | yes | no | yes |
| K225 | yes | yes | yes | no | yes | yes | yes | no | yes | no |
| K234 | yes | no | no | yes | yes | no | no | no | no | yes |
| K261 | yes | yes | no | no | yes | yes | no | no | no | no |
| K264 | yes | yes | no | yes | no | no | no | yes | no | no |
| K272 | yes | yes | no | yes | no | no | no | yes | no | no |
| K282 | yes | no | no | yes | yes | no | no | no | no | yes |
| K283 | yes |  |  |  |  |  |  |  |  |  |
| K284 | yes | no | yes | no | yes | no | no | no | yes | no |
| K290 | yes | no | no | no | yes | no | no | no | no | no |
| K294 | yes | yes | no | no | yes | yes | no | no | no | no |
| D013 | no | no | no | no | no | no | no | no | no | no |
| D027 | no | yes | no | yes | yes | yes | no | yes | no | yes |
| D029 | no | yes | no | no | no | no | no | no | no | no |
| D033 | no | yes | no | no | no | no | no | no | no | no |
| D044 | no | yes | no | no | no | no | no | no | no | no |
| D046 | no | no | no | no | yes | no | no | no | no | no |
| D049 | no | no | no | no | no | no | no | no | no | no |
| D053 | no | yes | no | no | no | no | no | no | no | no |
| D069 | no | no | no | no | yes | no | no | no | no | no |
| D074 | no | no | no | yes | yes | no | no | no | no | yes |
| D079 | no | yes | no | no | yes | yes | no | no | no | no |
| D083 | no | yes | no | yes | yes | yes | no | yes | no | yes |
| D100 | no | no | no | no | no | no | no | no | no | no |
|  |  |  |  |  |  |  |  |  |  |  |
|  |  |  |  |  |  |  |  |  |  |  |
